# An evaluation of KIF20A as a prognostic factor and therapeutic target for lung adenocarcinoma using integrated bioinformatics analysis

**DOI:** 10.3389/fbioe.2022.993820

**Published:** 2022-12-23

**Authors:** Dongjie Sun, Haiying Zhang, Chi Zhang, Lina Wang

**Affiliations:** ^1^ College of Basic Medical Sciences, Jilin University, Changchun, China; ^2^ Key Laboratory of Pathobiology, Ministry of Education, College of Basic Medical Sciences, Jilin University, Changchun, China; ^3^ Department of Anesthesiology, The First Hospital of Jilin University, Changchun, China; ^4^ Department of Pediatric Respiration, The First Hospital of Jilin University, Changchun, China

**Keywords:** lung adenocarcinoma, KIF20A, bioinformatics analysis, prognosis, therapeutic target

## Abstract

The identification of prognostic and therapeutic biomarkers is essential to reduce morbidity and mortality from lung adenocarcinoma (LUAD). This study aimed to identify a reliable prognostic and therapeutic biomarker for LUAD using integrated bioinformatics. Based on the cancer genome atlas (TCGA) and genome-tissue expression (GTEx) analyses, *KIF20A* has been identified as the hub gene. Following validation using a series of cohorts, survival analysis, meta-analysis, and univariate Cox analysis was conducted. ESTIMATE and CIBERSORT algorithms were then used to study the association of *KIF20A* with the tumor microenvironment (TME) and the percentage of tumor-infiltrating immune cells (TICs). *In vitro* experiments were conducted to determine the function of *KIF20A*. Finally, there was a negative association between the expression of the *KIF20A* and overall survival, progression-free survival, and disease-free survival, which was confirmed by meta-analysis and COX analysis. Furthermore, *KIF20A* also had a potential role of altering the TME and TICs proportions in LUAD. Validations *in vitro* were performed on A549 and PC-9 cell lines, and we found that the knockdown of KIF20A exhibited inhibitory effects on cell proliferation, resulted in cell cycle arrest during the G2/M phase, and induced cellular apoptosis. Our study demonstrated that *KIF20A* could be utilized as a reliable prognostic marker and treatment target for LUAD. However, further studies are required to validate these findings.

## 1 Introduction

Lung adenocarcinoma (LUAD) is the most common histological subtype of lung cancer. In the case of LUAD, the prognosis is extremely poor, with a five-year survival rate of less than 10% (Zhao et al., 2018; Fu et al., 2020). Various studies found that the aberrant expression of oncogenes, particularly those that drive cellular proliferation, increase resistance to therapy, and protect tumor cells against immune surveillance, play an essential role in tumor progression, subsequently leading to worse clinical outcomes (Zhu et al., 2015; Schabath et al., 2016; Chuang et al., 2017; Ma et al., 2019). Furthermore, tumor initiation and progression are influenced by the tumor microenvironment (TME), particularly the immune microenvironment (Gajewski et al., 2013; Hinshaw and Shevde, 2019). However, the mechanism by which the TME is involved in tumor development has not been elucidated. It is essential for the identification of potential oncogenes, alterations in the TME, and other cancer drivers to understand tumor pathogenesis, and accordingly, develop personalized, targeted therapies.

Kinesin family member 20A (KIF20A), also known as RAB6KIFL, belongs to the kinesin superfamily-6. There has been evidence that members of the superfamily are involved in vital cellular processes, such as spindle assembly, intracellular transport, and cellular mitosis (Shen et al., 2019; Xiong et al., 2019; Zhang et al., 2019). Furthermore, KIF20A dominantly functioned in chromosome partitioning and mitotic spindle formation (Xiong et al., 2019). Recent studies have demonstrated that *KIF20A* is overexpressed in many malignant tumors, including gastric cancer, glioma, bladder cancer, breast cancer, and prostate cancer (Li et al., 2019; Shen et al., 2019). Additionally, Shen et al. demonstrated that high KIF20A expression is associated with high tumor grade, advanced stage, and poor prognosis for bladder cancer patients (Shen et al., 2019). The prognostic significance of KIF20A has also been evaluated in other solid tumors, where it has been demonstrated that it plays a vital role in cell proliferation and tumor metastasis. However, there was limited knowledge about potential mechanisms of KIF20A in LUAD.

**FIGURE 1:**
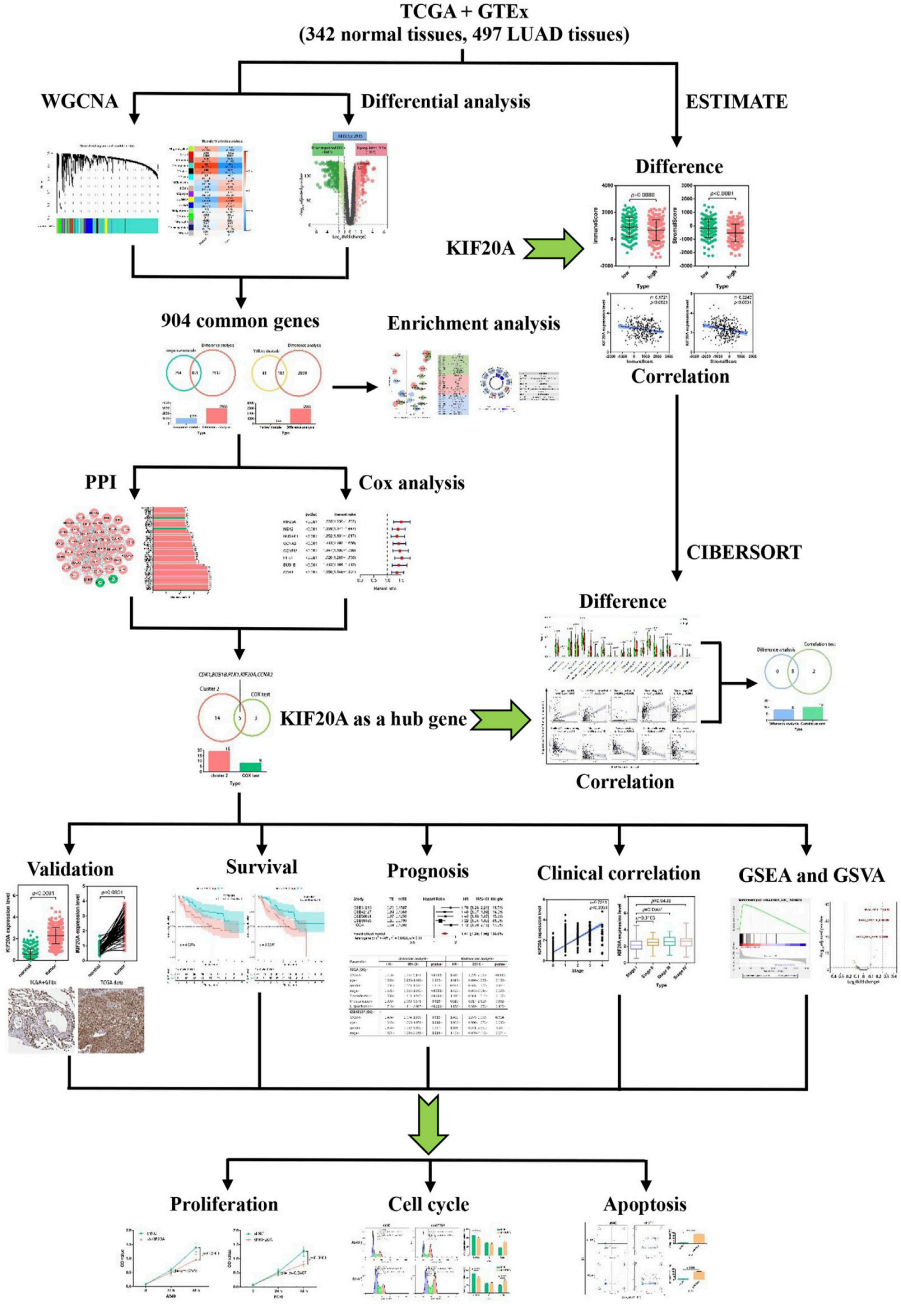
Flowchart for the work in lung adenocarcinoma (LUAD).

Integrative bioinformatics analysis combined with molecular biology and information technology is increasingly used to identify novel diagnostic and therapeutic biomarkers. This study aims to determine whether KIF20A can function as a prognostic and therapeutic biomarker for LUAD *via* integrated bioinformatics analysis. The flowchart of the work is shown in Figure 1.

## 2 Materials and methods

### 2.1 Data collection and preprocessing

RNA-sequence data for LUAD was extracted from The Cancer Genome Atlas (TCGA) database (https://portal.gdc.cancer.gov/), whereby 54 normal lung samples and 497 LUAD cases were identified. Another mRNA expression profile for 288 normal tissue samples was retrieved from the Genotype-Tissue Expression (GTEx) database (https://www.gtexportal.org/home). GSE19188 and GSE33532 profiles were downloaded from the Gene Expression Omnibus (GEO) database (https://www.ncbi.nlm.nih.gov/geo/). GSE19188 profile contained 65 normal samples and 91 LUAD samples. GSE33532 profile included 20 normal samples and 80 LUAD samples. The R package “limma” was used to combine TCGA and GTEx datasets for the following study.

### 2.2 Weighted gene co-expression network analysis and differential analysis

Co-expression network analyses using the R package “WGCNA” were conducted to detect high tumor-related modules and identify potential genes involved in these modules. This technique involved transforming the adjacency matrix into a topological overlap matrix (TOM) based on the soft threshold power. According to the TOM-based dissimilarity measure, the genes were then grouped into distinct modules. The cutoff threshold for the minModuleSize and mergeCutHeight were 30 and 0.25, respectively. Additionally, the genes with gene significance (GS) greater than 0.5 and a module membership (MM) above 0.8 were classified as candidate genes. The R package “edgeR” was used for differential expressed gene (DEG) identification between normal lung and LUAD cases. The parameters used for the DEGs analysis were an adjusted *p*-value < 0.5 and a |log_2_(fold change)| > 1. Finally, the candidate hub genes were codetermined by the promising genes in WGCNA and the DEGs analysis.

### 2.3 Function and pathway enrichment analysis

Gene oncology (GO) enrichment analysis was conducted using “clusterProfiler” in R, and candidate hub genes were determined by KEGG enrichment analyses. Statistical significance was defined as an adjusted *p*-value of less than 0.05. Gene set enrichment analysis (GSEA) and gene set variation analysis (GSVA) were used to identify the dominant pathways associated with the hub genes. The “c2.cp.kegg.v7.1.symbols.gmt” gene set was identified from the molecular signature database (MSigDB) (http://software.broadinstitute.org/gsea/msigdb/index.jsp). Pathway expression was profiled using the R package “pheatmap”.

### 2.4 Protein-protein interaction (PPI) construction network and COX analysis

For the candidate hub genes, the interaction information among proteins with a combined score above 0.9 was retrieved using the retrieval of interacting genes database (STRING) search tool (https://string-db.org/). To visualize the PPI network and carry out a clustering analysis of the network, we used Cytoscape software (version 3.6.0) and its molecular complex detection plugin (MCODE). For the top two clusters with the highest MCODE scores, the connectivity degrees of the nodes were calculated. Moreover, the R package “survival” was adopted to conduct univariate COX regression analysis for the corresponding genes within the two clusters. The common DEGs overlapped of nodes with connectivity degrees above 30 in the clustering network and a *p*-value below 0.001 in the COX analysis were identified as hub genes.

### 2.5 Hub gene validation

A quantitative real-time polymerase chain reaction (qRT-PCR) was used to measure the expression of hub genes. In addition, the hub gene was validated through several differential analyses utilizing the TCGA and GTEx data and the profiles GSE19188 and GSE33532. Further validation was performed using the Oncomine meta-analysis platform (https://www.oncomine.org). The protein expression of the corresponding gene was also examined using Western blot analysis, differential analysis based on the Clinical Proteomic Tumor Analysis Consortium (CPTAC) database (https://cptac-data-portal.georgetown.edu/), and immunohistochemical analysis of images obtained from the Human Protein Atlas (HPA) database (https://www.proteinatlas.org).

### 2.6 Survival analysis and meta-analysis

We used the R packages “survival” and “survminer” to analyze the correlation between *KIF20A* expression and overall survival (OS) as well as progression-free survival (PFS) among patients with LUAD. The survival analyses for *KIF20A* were also conducted utilizing the Gene Expression Profiling Interactive Analysis (GEPIA) database (http://gepia.cancer-pku.cn) and Kaplan-Meier plotter platform (https://kmplot.com/analysis/). Meta-analysis of the five cohorts was conducted using R packages “survival” and “meta”. Pearson’s method was used to analyze the correlation between each term using the GraphPad Prism software (version 7.0).

### 2.7 Association between hub gene and tumor microenvironment (TME)

Using the “ESTIMATE” package in R, the proportion of immune and stromal cells in LUAD tissues was calculated. The total ESTIMATE score was calculated by combining the two scores. Based on the CIBERSORT algorithm, we determined the relative community of tumor-infiltrating immune cells (TICs). The R packages “circlize” and “corrplot” were developed to visualize the correlation among TICs.

### 2.8. Cell culture and plasmid transfection

We obtained the cell lines HBE, A549, PC-9, and NCI-H1395 from the Shanghai Cell Bank of the Chinese Academy of Medical Sciences (Shanghai, China). Cells were cultured in high glucose Dulbecco’s Modified Eagle’s medium (DMEM; Hyclone, Logan, Utah, United States) containing 10% fetal bovine serum (FBS; Gibco, Grand Island, NY, United States) and 1% penicillin-streptomycin (MRC, Jintan, China) at 37°C and 5% Carbon dioxide (CO_2_). The *KIF20A*-targeted sequence of short hairpin RNAs (shRNA, 5′- GCC​ACT​CAC​AAA​TTT​ACC​TTT-3′) and the scrambled sequence (5′-CCT​AAG​GTT​AAG​TCG​CCC​TCG-3′) were cloned into pLKO.1 plasmid. Both pLKO.1-KIF20A-shRNA (shKIF20A) and pLKO.1-scramble-shRNA (shNC) were provided by the Chinese Public Protein Plasmid Library (PPL, Nanjing, China). A549 and PC-9 cells were transfected using the X-tremeGENE HP DNA transfection reagent (Roche, Shanghai, China) according to the manufacturer’s guidelines.

### 2.9 Quantitative real-time PCR analysis

The Total RNA Extraction Kit (Solarbo, Beijing, China) was used to isolate the total RNA. First-strand cDNA synthesis kit (Invitrogen, Carlsbad, CA, United States) and Premix Ex Taq SYBR Green PCR kit (Takara, Dalian, China) were used according to the manufacturer’s protocols for reverse transcription and quantitative PCR, respectively. The primers were synthesized as follows: *KIF20A*, forward: 5′-TGC​TGT​CCG​ATG​ACG​ATG​TC-3′ and reverse: 5′-AGG​TTC​TTG​CGT​ACC​ACA​GAC-3'; *GAPDH*, forward: 5′-GGA​GCG​AGA​TCC​CTC​CAA​AAT-3′ and reverse: 5′-GGC​TGT​TGT​CAT​ACT​TCT​CAT​GG-3'.

### 2.10 Western blot analysis

Extraction and quantification of total protein were conducted. Six percent SDS-PAGE gels were then used to separate and transfer the proteins onto polyvinylidene fluoride membranes. Membranes were blocked with five percent skimmed milk for two hours at room temperature and then incubated with the primary antibody against KIF20A (ThermoFisher, Waltham, MA, United States, 1:1000 dilution, Catalog #PA5-83359) and β-actin (Abcam, Cambridge, United Kingdom, 1:1000 dilution, ab8226) overnight at four degrees. Thereafter, the bands were incubated with a horseradish peroxidase-conjugated secondary antibody (Bioss, Beijing, China) at room temperature for an hour and were subsequently visualized using enhanced chemiluminescence reagents (Beyotime, Shanghai, China).

### 2.11 Assessment of cell proliferation

Cell counting kit (CCK)-8 kit (Beyotime) and colony formation assay were conducted to assess cell proliferation ability. A total of 6,000 cells were cultured overnight in 96-well plates. On the following day, cells were transfected with the corresponding plasmid. The CCK-8 solution was added to each well after 24–48 h to determine the cells’ viability. The colony formation assay was performed by culturing 1,000 cells per well overnight in six-well plates, followed by transfection. A new culture medium was added the next day, and the cells were kept in culture for an additional 14 days. The colonies were then stained with Giemsa solution (Beyotime) and calculated using ImageJ (version 1.8.0).

### 2.12. Cell cycle and apoptosis assessment

Overnight at 4°C, the transfected cells were fixed in 70% ethanol. Afterward, the cells were stained with 500 μL PI/RNase staining buffer (BD Pharmingen, San Diego, CA, United States) for 15 min at 37°C or else incubated with 5 μL FITC Annexin V (BD Pharmingen), 5 μL propidium iodide (PI, BD Pharmingen) and 400 μL binding buffer for 15 min at 25°C in the dark. A flow cytometer (FCM) (BD FACSVerse, San Jose, CA, United States) was used to analyze the cell cycle and apoptosis according to the manufacturer’s instructions.

### 2.13 Statistical analysis

GraphPad Prism (version 7.0) and R software (version 3.6.0) were used for statistical analysis. Log-rank tests were used to calculate the statistical significance of the survival analysis. In the meta-analysis, heterogeneity was measured using Cochran’s Q test and Higgin’s *I* ^*2*^ statistics. The results of qRT-PCR were analyzed by the 2^-△△Ct^ method. Student’s t-tests and one-way analyses of variance (ANOVA) were used to determine whether there was a significant difference between LUAD and normal cells. Experiments were conducted at least three times, and the quantitative data are presented as mean ± standard deviation (SD). *p*-values below 0.05 were considered statistically significant.

## 3 Results

### 3.1 Identification of candidate hub genes

**FIGURE 2:**
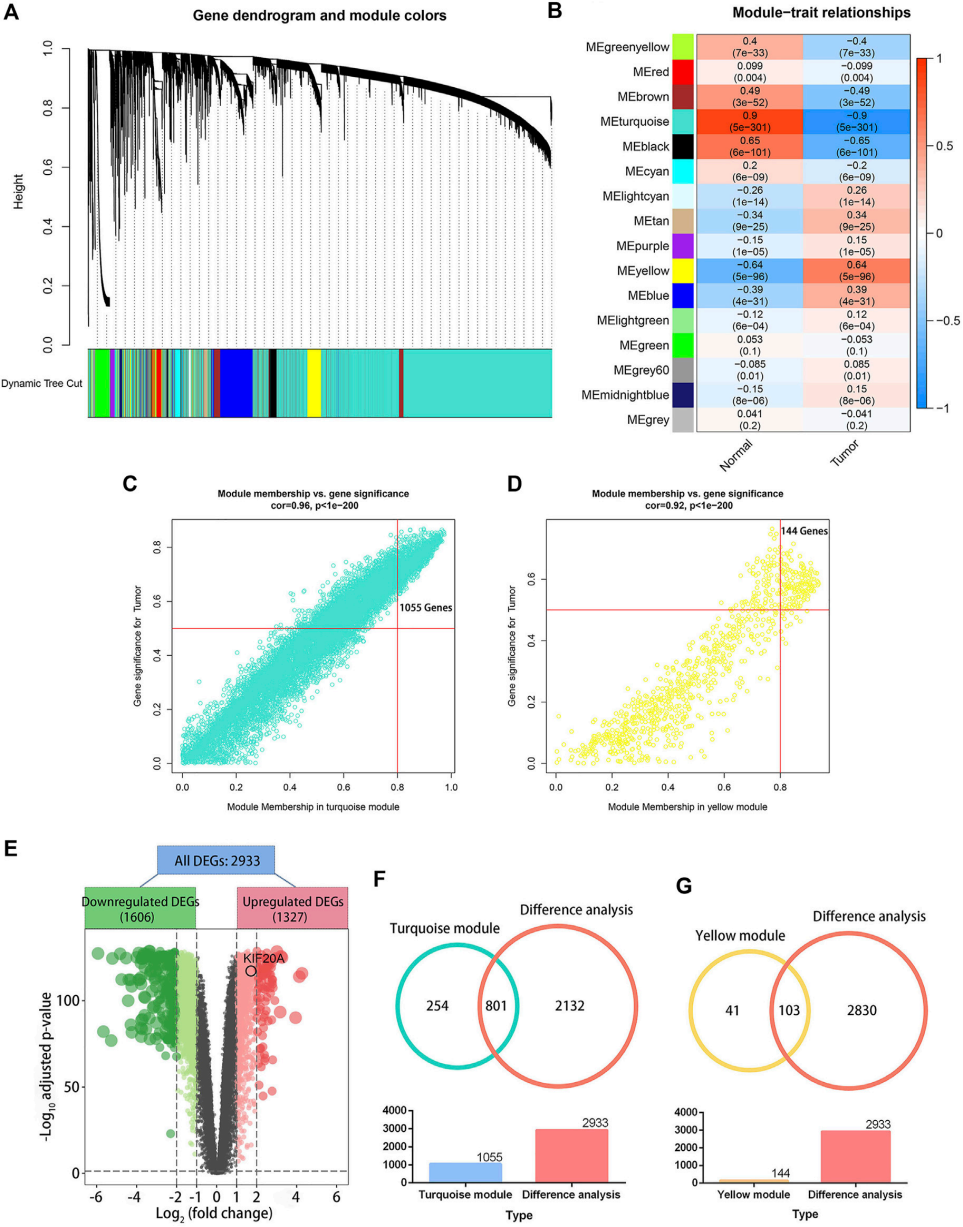
Weighted gene co-expression network analysis (WGCNA) and differential analysis. Image **(A)** illustrates a clustering dendrogram for the GTEx and TCGA datasets based on the dissimilarity measure (1-TOM). Heatmap **(B)** shows the correlation between module eigengenes and clinical traits in LUAD. The scatter plots illustrate the eigengenes for the turquoise **(C)** and yellow **(D)** modules. A module membership above 0.8 and a gene significance greater than 0.5 were considered statistically significant. The volcano plot **(E)** shows 2,933 differentially expressed genes (DEGs) in LUAD samples *versus* normal lung tissues. An adjusted *p*-value < 0.05 and |log_2_(fold change)| > 1 were set as the cutoff criterion. The Venn plots **(F)** illustrate 801 genes overlapped by the turquoise module eigengenes and differential analysis, and the 103 genes were codetermined by the yellow module eigengenes and differential analysis **(G)**.

A total of 839 samples (342 normal samples and 497 LUAD samples) were clustered according to their mRNA expression similarity. No outliers within the samples were removed, and the distribution of clinical traits within the samples was shown in Supplementary Figure S1. In the WGCNA, a soft-thresholding power (*β*) of five and a scale-free R^2^ of 0.95 were adopted for a scale-free network, as shown in Supplementary Figure S2. Based on this method, 16 modules were identified, as shown in Figure 2A. The turquoise module showed the strongest negative association with tumor samples, as shown in Figure 2B, and had a higher module significance (MS) than the other modules, as shown in Supplementary Figure S3. In general, MS was defined as the average gene significance (GS) for all genes within a particular module, indicating the overall correlation between a trait and the module. Additionally, the yellow module was the most positively associated with tumor samples. There were 1,055 and 144 genes with GS > 0.5 and MM > 0.8, respectively, in the turquoise and yellow modules, as shown in Figure 2C and Figure 2D. The differential analysis between normal and LUAD tissues revealed a total of 2,933 DEGs, containing 1,327 upregulated DEGs and 1,606 downregulated DEGs, as shown in Figure 2E. There were 801 common genes between all DEGs and turquoise module-related genes and 103 common genes between DEGs and yellow module-related genes, as shown in Figure 2F and Figure 2G. The 904 intersected DEGs were defined as candidate hub genes and used for the following analysis.

### 3.2 GO and KEGG enrichment analyses

**FIGURE 3:**
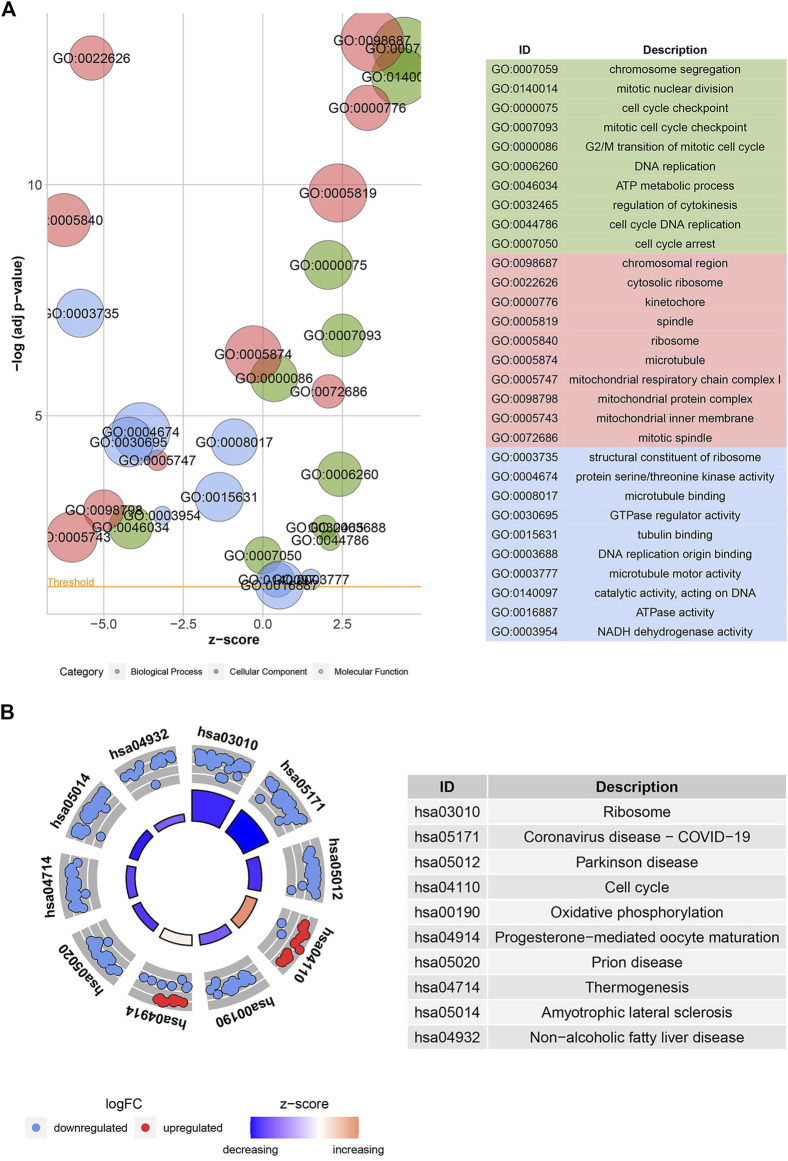
Gene ontology (GO) and Kyoto encyclopedia of genes and genomes (KEGG) enrichment analyses. Bubble plot **(A)** shows the GO function analysis for the 904 common DEGs. The circle plot **(B)** shows the KEGG pathway analysis for the common DEGs. The higher the z-score, the higher the expression of enriched terms.

According to GO analysis, the 904 common DEGs were mainly enriched in chromosome segregation, mitotic nuclear division, cell cycle checkpoint, and other biological processes, as shown in Figure 3A. The chromosomal region, cytosolic ribosome, and kinetochore were the primarily enriched cellular component terms. For molecular function enrichment, DEGs were mainly involved in the structural constituent of the ribosome, protein-threonine kinase activity, microtubule-binding, and other terms. More detailed information is provided in Supplementary Table S1. In addition, KEGG analysis revealed that DEGs were predominantly associated with the ribosome, cell cycle, oxidative phosphorylation, and other biological pathways, as shown in Figure 3B. The information is further illustrated in Supplementary Table S2.

### 3.3 *KIF20A* was identified as a hub gene

**FIGURE 4:**
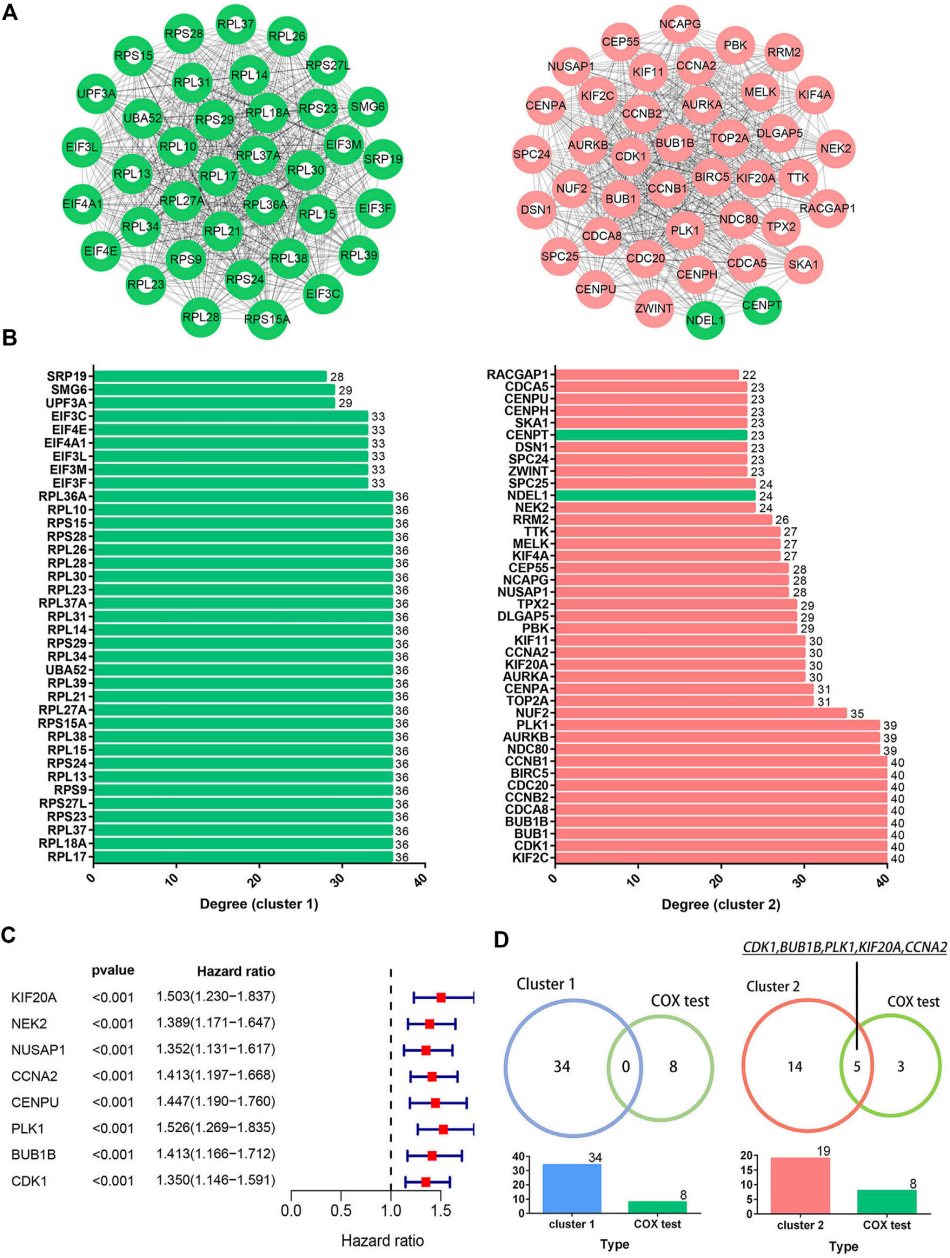
Identification of hub genes. Image **(A)** illustrates the network of the top two clusters in the protein-protein interaction network containing 904 common DEGs. Green and red colored spots indicate downregulated and upregulated DEGs, respectively. Image **(B)** illustrates the degrees of connectivity within the nodes in the corresponding cluster networks. Image **(C)** shows the results of the univariate COX analysis for the significant factors in selected clusters (*p* < 0.001). Image **(D)** shows the five common genes overlapped by the nodes with connectivity degrees above 30 in the cluster network (n > 30) and a *p*-value below 0.001 in the COX analysis.

PPI network constructed from candidate DEGs contained 391 nodes and 2,794 edges, as shown in Supplementary Figure S4. Meanwhile, the top six clusters in the PPI network were developed *via* the MCODE plugin, as shown in Table 1, of which clusters one and two had the highest MCODE scores and were therefore selected as the key clusters, as shown in Figure 4A. The connectivity degrees of the nodes within key clusters were calculated and visualized in the histograms, as shown in Figure 4B. Among the DEGs in key clusters, univariate COX analysis was carried out to determine the significant factors (*p* < 0.001) for the overall survival (OS) in LUAD patients, as shown in Figure 4C. According to COX analysis, five common genes, including *CDK1*, *BUB1B*, *PLK1*, *KIF20A*, and *CCNA2*, were shared by leading nodes with a connectivity degree above 30 and a *p*-value below 0.001 in the second cluster. However, no genes were identified in the first cluster, as shown in Figure 4D. Amongst the five common factors, *KIF20A* was further evaluated and classified as the hub gene for the following study.

**TABLE 1:** The top six MCODE clusters in the PPI network.

MCODE cluster	Nodes	Edges	MCODE scores	MCODE cluster	Nodes	Edges	MCODE scores
cluster 1	37	646	36.541	cluster 4	12	66	12.000
cluster 2	41	625	31.250	cluster 5	9	36	9.000
cluster 3	12	66	12.000	cluster 6	9	36	9.000

PPI, protein-protein interaction; MCODE, molecular complex detection.

### 3.4 *KIF20A* exhibited high expression in LUAD tissues

**FIGURE 5:**
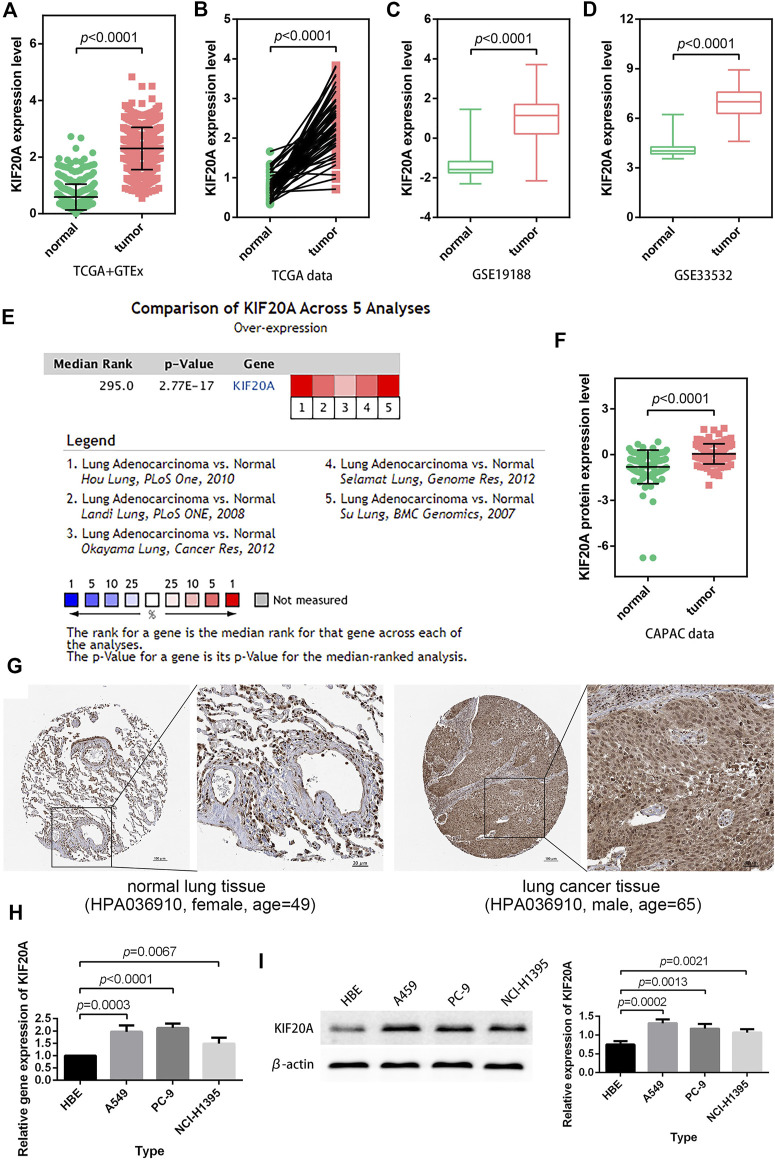
Verification of *KIF20A* expression. LUAD samples had higher KIF20A expression in both unpaired **(A)** and paired **(B)** differential analyses, and the observations were verified using GSE19188 **(C)** and GSE33532 **(D)** profiles. Meta-analysis **(E)**, protein differential analysis **(F)**, and immunohistochemical images **(G)** validated the overexpression of the *KIF20A* gene in LUAD. Quantitative real-time PCR (qRT-PCR) **(H)** and Western blot analysis **(I)** illustrate the significant upregulation of*KIF20A* expression in A549, PC-9, and NCI-H1395 cells compared with HBE cells.

In LUAD samples, KIF20A expression was significantly higher than in normal samples, as shown in Figures 5A–D. According to a meta-analysis using the Oncomine database, LUAD tissues had a higher expression of *KIF20A* than normal lung tissues in five cohorts (Hou lung, Landi lung, Okayama lung, Selamat lung, and Su lung), as shown in Figure 5E (Hou et al., 2010; Landi et al., 2008; kayama et al., 2012; Selamat et al., 2012; Su et al., 2007). Furthermore, higher levels of KIF20A proteins were detected in LUAD tissues as compared with normal lung tissues, as shown in Figure 5F. The observation that *KIF20A* had increased expression in LUAD was further assessed in A549, PC-9, and NCI-H1395 cell lines by qRT-PCR and Western blot analyses, as shown in Figure 5H.

### 3.5. Identification of *KIF20A* as a prognostic factor

**FIGURE 6:**
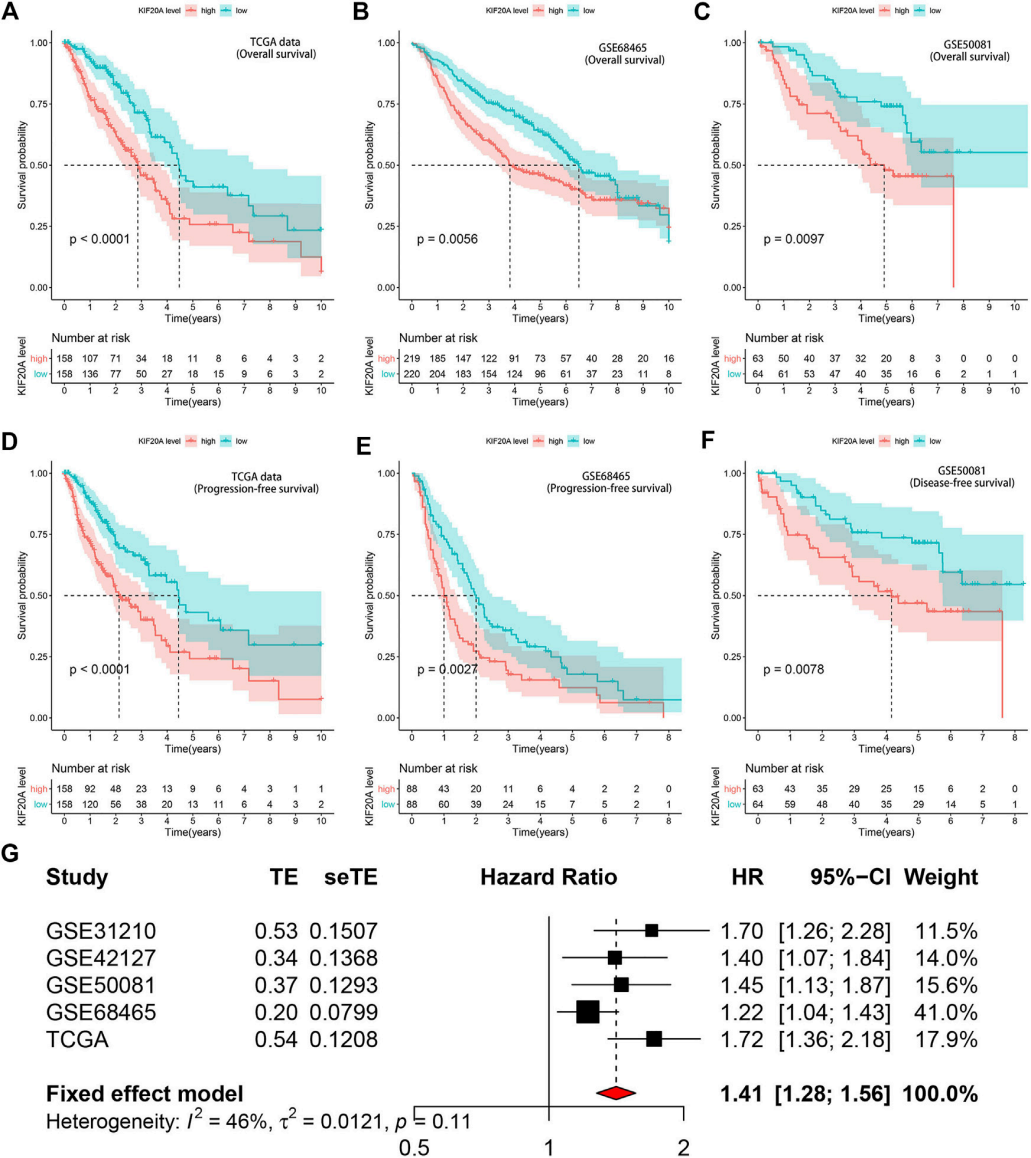
Prognostic value of KIF20A expression in LUAD. Kaplan-Meier survival analysis showed *KIF20A* expression significantly indicated worse OS in LUAD patients in the TCGA dataset **(A)**, GSE68465 **(B)**, and GSE50041 **(C)** profiles. Kaplan-Meier survival analysis showed that KIF20A expression was negatively associated with progression-free survival (PFS) in the TCGA dataset **(D)** and GSE68465 profile **(E)**, as well as disease-free survival (DFS) in the GSE50081 profile **(F)**. The meta-analysis **(G)** further confirmed the correlation of *KIF20A* expression with OS in the five cohorts.

Kaplan-Meier survival analysis revealed that high *KIF20A* expression is a significant indicator of poor OS and unfavorable PFS in LUAD patients, as shown in Figures 6A–E. Besides, *KIF20A* showed a negative association with DFS in the GSE50081 profile, as shown in Figure 6F. These were further confirmed in the GEPIA database and Kaplan-Meier Plotter platform, as shown in Supplementary Figure S5. As there was no heterogeneity among the five datasets (*I* ^*2*^ < 50% and *p* > 0.05), we chose a fixed model to conduct the meta-analysis. The results of the meta-analysis suggested that *KIF20A* served as a high-risk factor for the survival of LUAD patients (HR = 1.41, 95% CI: 1.28–1.56), as shown in Figure 6G. Subsequently, univariate Cox analysis demonstrated that *KIF20A* had a significantly negative correlation with the survival of LUAD patients in several datasets, as shown in Table 2. According to the above results, KIF20A may serve as a prognostic factor for LUAD patients.

**TABLE 2:** Univariate Cox regression analysis for *KIF20A* expression on OS, PFS, and DFS in LUAD patients.

Parameter	Univariate analysis			Multivariate analysis		
	HR	95% CI	P-value	HR	95% CI	P-value
TCGA (OS)						
KIF20A	1.718	1.356–2.178	<0.001	1.685	1.299–2.185	<0.001
age	1.000	0.983–1.019	0.958	1.014	0.996–1.033	0.133
gender	1.091	0.774–1.539	0.618	0.971	0.685–1.377	0.87
stage	1.581	1.350–1.852	<0.001	1.422	0.943–2.145	0.093
T classification	1.598	1.313–1.945	<0.001	1.205	0.961–1.512	0.107
M classification	1.900	1.069–3.378	0.029	0.910	0.317–2.618	0.862
N classification	1.716	1.411–2.087	<0.001	1.156	0.810–1.650	0.425
GSE42127 (OS)						
KIF20A	1.404	1.074–1.835	0.013	1.452	1.079–1.953	0.014
age	1.043	1.008–1.079	0.016	1.032	0.996–1.070	0.085
gender	1.804	0.959–3.392	0.067	1.308	0.671–2.551	0.43
stage	1.571	1.094–2.256	0.014	1.431	0.970–2.110	0.071
TCGA (PFS)						
KIF20A	1.876	1.463–2.407	<0.001	1.788	1.366–2.340	<0.001
age	1.001	0.984–1.019	0.868	1.013	0.995–1.031	0.162
gender	1.127	0.800–1.589	0.494	1.024	0.723–1.449	0.895
stage	1.573	1.339–1.847	<0.001	1.394	0.935–2.076	0.103
T classification	1.592	1.318–1.923	<0.001	1.235	0.989–1.543	0.062
M classification	2.053	1.151–3.662	0.015	0.939	0.342–2.582	0.903
N classification	1.603	1.319–1.948	<0.001	1.054	0.749–1.483	0.764
GSE68465 (PFS)						
KIF20A	1.460	1.153–1.848	0.002	1.403	1.070–1.841	0.014
gender	1.225	0.871–1.721	0.243	1.186	0.840–1.673	0.333
age	1.010	0.991–1.030	0.301	1.016	0.997–1.036	0.106
grade	1.492	1.125–1.979	0.006	1.169	0.863–1.585	0.313
N classification	1.575	1.267–1.959	<0.001	1.582	1.255–1.995	<0.001
T classification	1.497	1.174–1.908	0.001	1.318	1.020–1.703	0.035
GSE50081 (DFS)						
KIF20A	1.414	1.105–1.809	0.006	1.339	1.025–1.751	0.033
age	1.018	0.989–1.047	0.238	1.017	0.985–1.049	0.296
gender	1.480	0.846–2.589	0.170	1.565	0.880–2.783	0.128
stage	2.514	1.415–4.465	0.002	2.191	0.357–13.435	0.397
T classification	2.749	1.461–5.173	0.002	1.697	0.799–3.606	0.169
N classification	2.219	1.236–3.982	0.008	0.984	0.163–5.935	0.986

LUAD, lung adenocarcinoma; HR, hazard ratio; CI, confidence interval; OS, overall survival; PFS, progress-free survival; DFS, disease-free survival.

### 3.6 Prognostic significance of *KIF20A* in LUAD

**FIGURE 7:**
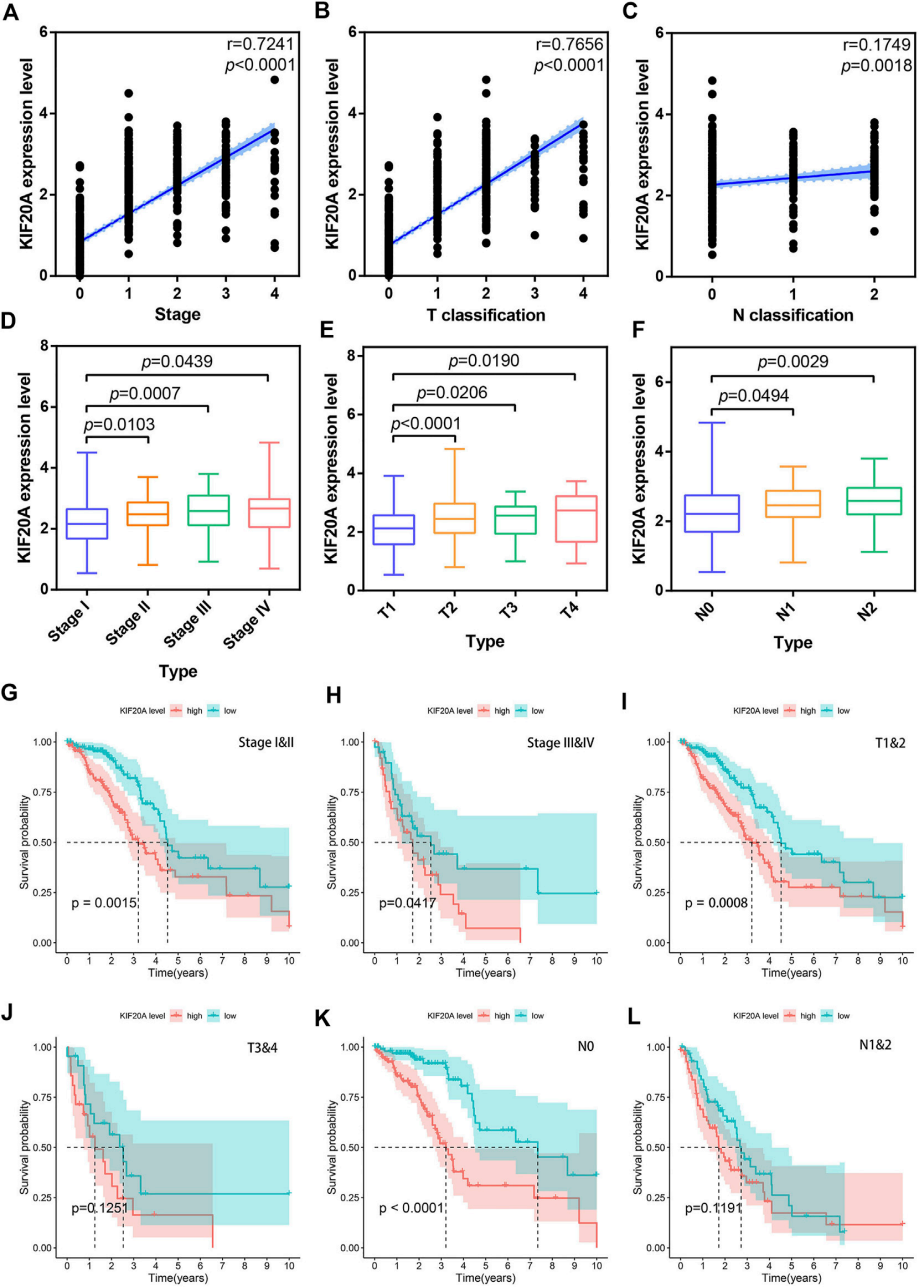
Correlation of *KIF20A* expression with clinical characteristics according to the TCGA dataset. Scatter plots show the correlation of *KIF20A* expression with tumor stage **(A)**, T classification **(B)**, and N classification **(C)**. Boxplots show the differential analysis of *KIF20A* expression in different tumor stages **(D)**, T classification **(E)**, and N classification **(F)**. Kaplan-Meier survival analysis shows the correlation of *KIF20A* expression with OS in LUAD patients according to tumor stage: stage I&II **(G)** and stage III and IV **(H)**, primary tumor: T1 and 2 **(I)** and T3 and 4 **(J)**, and lymph node metastasis: N0 **(K)** and N1&2 **(L)**.

Based on TCGA data, both correlation test and differential analysis indicated that *KIF20A* had a dominantly positive association with T-stage and N-stage in LUAD patients, as shown in Figures 7A–F. Still, there was no significant difference in KIF20A expression based on age (above or below 65), gender (female and male), and distant metastasis, as shown in Supplementary Figure S6. Additionally, *KIF20A* significantly predicted poor OS in LUAD patients with certain clinical characteristics; stage I and II (*p* = 0.0015), stage III and IV (*p* = 0.0417), T1 and T2 (*p* = 0.0008), and N0 (*p* < 0.0001), but not in LUAD patients with T3 and T4 (*p* = 0.1251) and N1 and N2 (*p* = 0.1191), as shown in Figures 7G–L.

### 3.7 *KIF20A* was involved in the cell cycle

**FIGURE 8:**
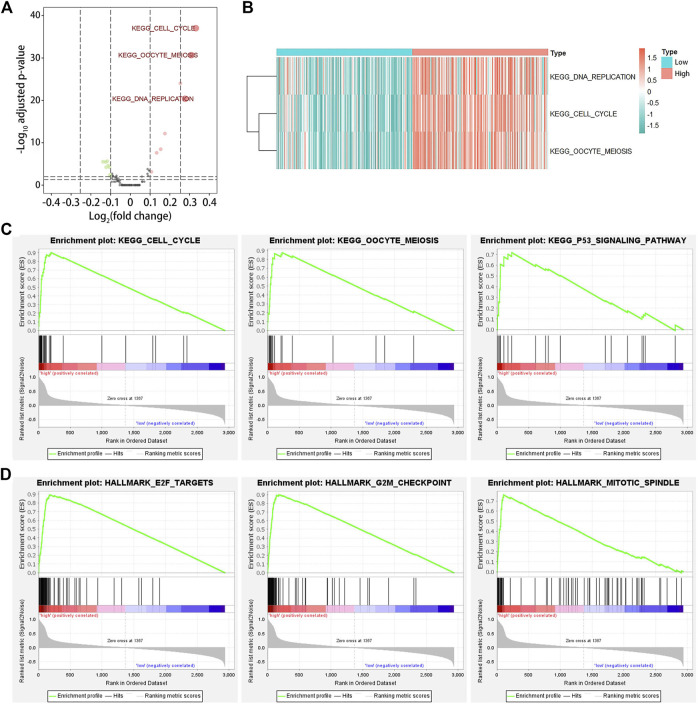
Gene set variation analysis (GSVA) and gene set enrichment analysis (GSEA). Image **(A)** illustrates the identification of differentially expressed pathways among LUAD patients by GSVA. LUAD patients were divided into low and high *KIF20A* expression subgroups based on the median *KIF20A* expression level. An adjusted *p*-value < 0.05 and log_2_|fold change| > 0.25 were set as the cutoff criterion. Image **(B)** shows the clustering heatmap for the differentially expressed pathways. Image **(C)** shows the enriched gene sets in KEGG, while image **(D)** shows the HALLMARK collections in the high *KIF20A* expression samples.

To identify the biological functions of *KIF20A* in LUAD, we conducted GSVA and GSEA for the DEGs between high and low KIF20A expression subgroups according to the median expression of *KIF20A*. The results of GSVA found that cell cycle pathways, oocyte meiosis, and DNA replication were markedly upregulated in high KIF20A expression samples compared with low KIF20A expression samples, as shown in Figure 8A. The results of GSEA found that oocyte meiosis and p53 signaling pathway were the mainly enriched cell cycle pathways in the KEGG collection and the E2F targets, G2M checkpoint, and mitotic spindle in the HALLMARK gene set collection, as shown in Figure 8C, Figure 8D, and Supplementary Table S3. The above results suggest that *KIF20A* was mainly associated with cell cycle in LUAD progression.

### 3.8 *KIF20A* was associated with TME

**FIGURE 9:**
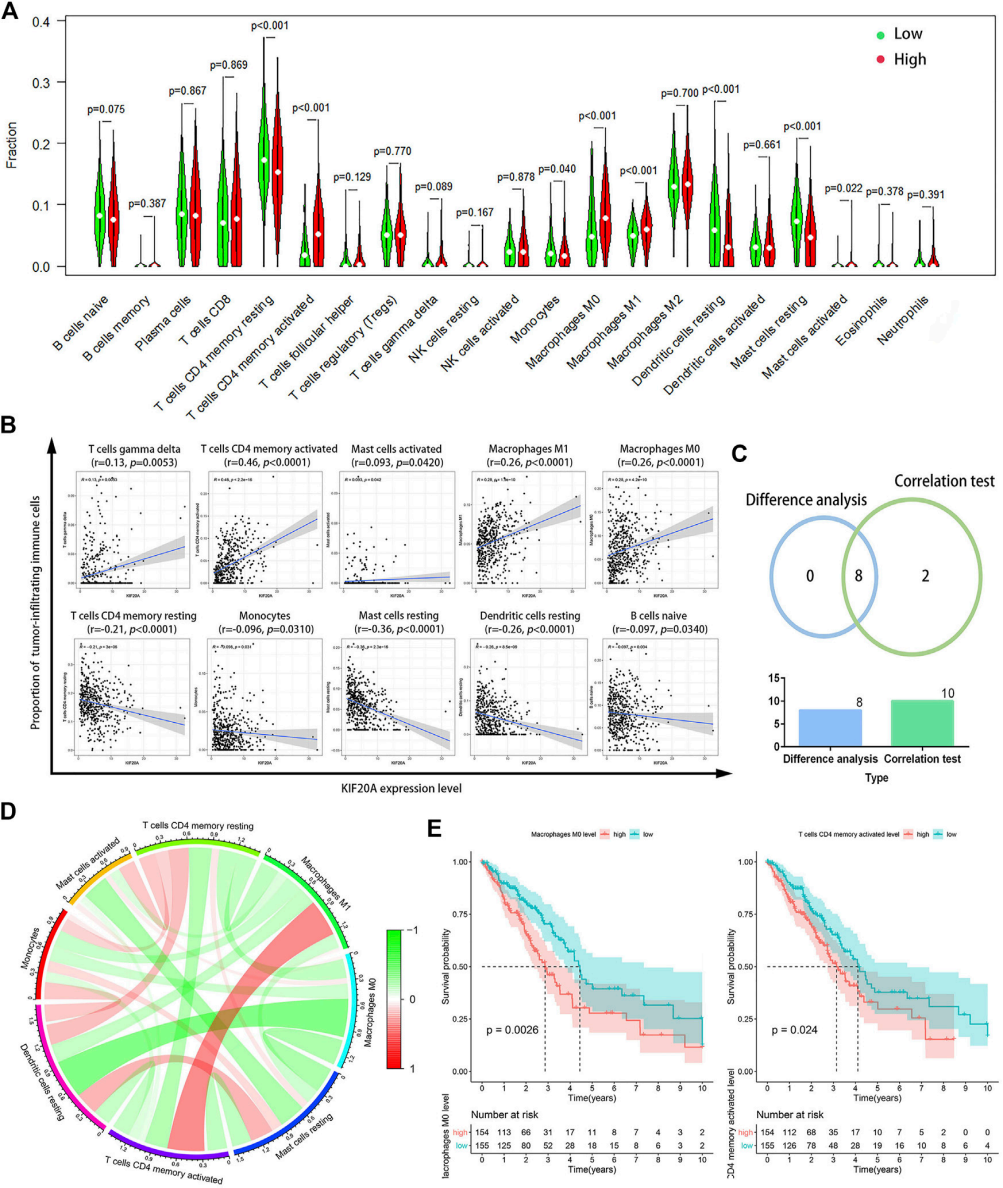
Correlation of *KIF20A* expression with tumor-infiltrating immune cells (TICs) proportion. Differential analysis **(A)** for the proportions of 21 kinds of TICs in LUAD samples with low or high *KIF20A* expression levels relative to the median *KIF20A* expression level. Scatter plots **(B)** show the correlation between the levels of the ten kinds of TICs and the *KIF20A* expression (*p* < 0.05). Venn diagram **(C)** shows the eight kinds of TICs correlated with *KIF20A* expression overlapped by the differential analysis and correlation test. Circle plot **(D)** shows the correlation among the eight different types of TICs. Image **(E)** illustrates the Kaplan-Meier overall survival (OS) analysis for the proportions of two types of TICs (*p* < 0.05).

Supplementary Figure S7 showed that *KIF20A* had a significant negative association with the immune score, stromal score, and total ESTIMATE score. CIBERSORT analysis results revealed eight TICs among 21 kinds of TICs prominently associated with *KIF20A*, codetermined by the differential analysis in the histogram and the correlation test scatter plot, respectively, as shown in Figures 9A–C. The correlation among the eight different types of TICs, as well as the association of eight TICs with OS, shows that levels of uncommitted macrophages (M0) and activated CD4 memory T-cells had a distinctively negative association with OS in patients with LUAD, as shown in Figure 9D, Figure 9E and Supplementary Figure S8.

### 3.9 *KIF20A* knockdown inhibited cell proliferation and induced cell cycle arrest

**FIGURE 10:**
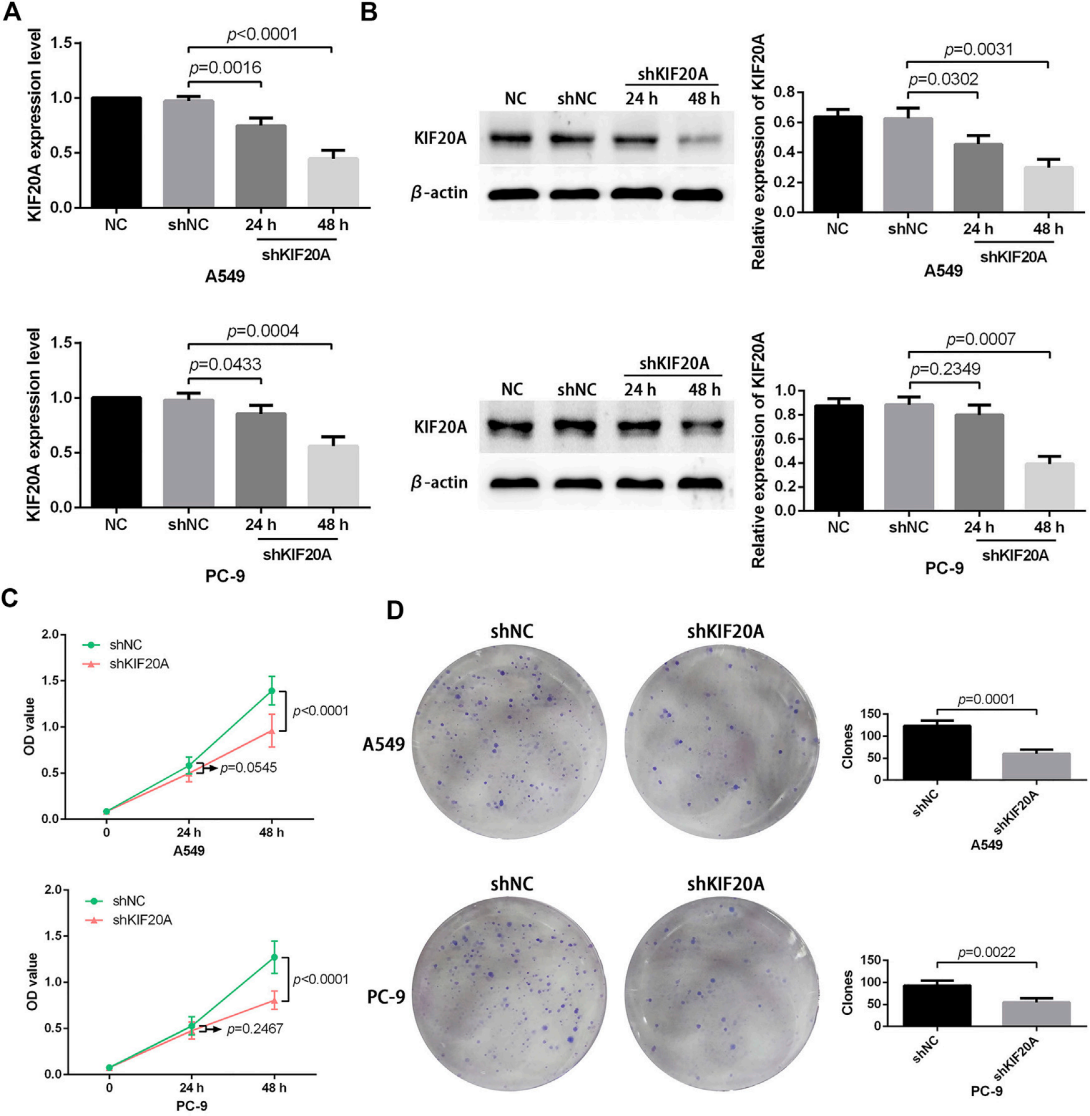
Effects of *KIF20A* knockdown on the proliferation of A549 and PC-9 cells. Efficacy of *KIF20A* deletion measured by qRT-PCR **(A)** and Western blot analysis **(B)**. *KIF20A* deletion inhibited cell growth in the CCK-8 test **(C)** and colony formation assay **(D)**.

**FIGURE 11:**
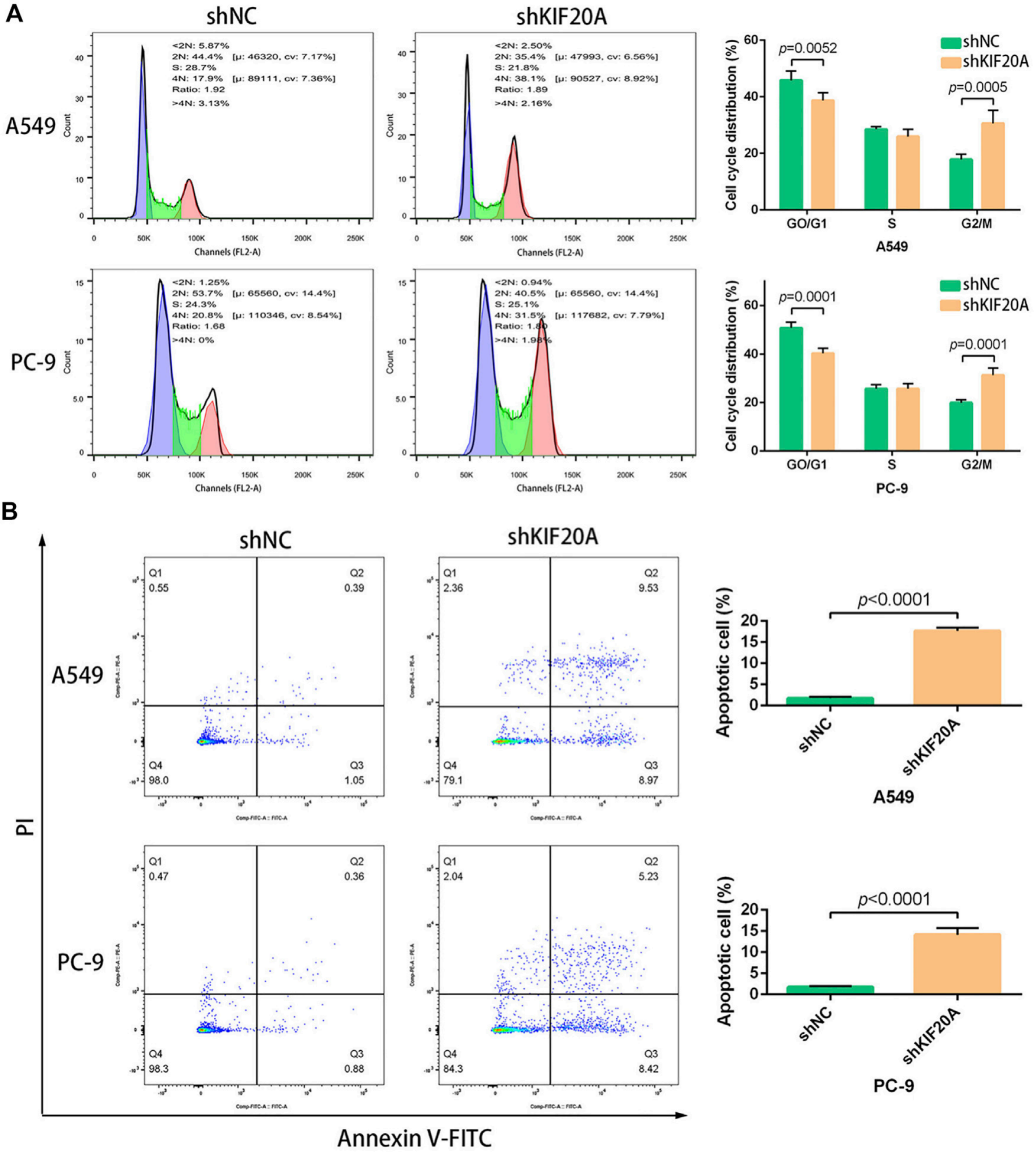
Effects of *KIF20A* knockdown on cell cycle and apoptosis inA549 and PC-9 cells. *KIF20A* knockdown induced G2/M arrest **(A)** and promoted apoptosis **(B)** 48 h after transfection.

Compared with shNC group, the shKIF20A efficiently reduced the expression of *KIF20A* in A549 and PC-9 cells after transfection, as shown in Figure 10A. At 48 h after the transfection, the shKIF20A group had lower cell viability than the shNC group, as shown in Figure 10C. The number of colonies was distinctively reduced in the shKIF20A group compared to the shNC group, as shown in Figure 10D. The FCM results demonstrated that the cell numbers of the G0/G1 phase were remarkably decreased 48 h after transfection, while the cell proportions at the G2/M phase were dominantly increased, as shown in Figure 11A. Moreover, the higher percentage of apoptosis was significantly associated with shKIF20A transfection, as shown in Figure 11B.

## 4 Discussion

Because of cancer metastasis and recurrence, LUAD has posed a significant obstacle to clinical outcomes. Accumulating findings indicate that multiple signaling pathways are activated during LUAD progression, including the MAPK signaling pathway, PI3K/AKT signaling axis, FOXM1 signals, WNT signaling pathway, JAK/STAT pathways, and others (Zhan et al., 2017; Gao et al., 2018; Hou et al., 2019; Shan et al., 2019; Sun and Zhao, 2019). These pathways have been found to contribute to tumor development by inducing cell cycle arrest at the resting-first growth (G0/G1) transition phase or the second growth mitosis transition (G2/M) phase. The FOXO3/FOXM1 axis is an essential downstream signal of the PI3K/AKT, Ras/ERK, and JNK/p38MAPK pathways, which are also involved in the tumor growth mediated by controlling the cell cycle (Yao et al., 2018). From these observations, we can conclude that the cell cycle plays an essential role in controlling tumor growth, suggesting that future therapeutic strategies for LUAD could target the key molecules involved in the cell cycle. In addition, numerous studies have shown that immunotherapy can potentially suppress tumor growth and metastasis by triggering the innate and adaptive immune systems (Farhood et al., 2019; Wculek et al., 2020). Studies have shown that the initiation and recurrence of LUAD are not only influenced by the oncogenic properties of cancer but also by its interaction with the TME, particularly the immune microenvironment.

In this study, we have identified *KIF20A* as the hub gene *via* integrated bioinformatics analysis. This gene was linked with a worse OS and could be used as a prognostic indicator in LUAD patients. Furthermore, it was also linked with alterations in the TME and TICs communities. The *KIF20A* knockdown was also found to inhibit LUAD cell proliferation, arrest cells at the G2/M phase, and promote apoptosis. *KIF20A*, also known as *MKLP2* and *RAB6KIFL*, is a type of protein belonging to the kinesin family. Most members of the kinesin family are characterized by conserved kinetic domain and ATP activity (Li et al., 2019; Xiong et al., 2019). The characteristics prompt them to participate in chromosome partitioning, cellular transport, and spindle formation. *KIF20A* was mainly involved in organelle dynamics and cell mitosis (Fontijn et al., 2001; Zhang et al., 2020). Aberrant expression of *KIF20A* might result in abnormal cellular behaviors in cancer cells. Xiong et al. distinctively demonstrated that *KIF20A* was highly expressed in colorectal cancer and was involved in pathological functions, including promoting cellular growth and increased resistance to chemotherapy, mediated by triggering the JAK/STAT3 signaling pathway. Li et al. indicated that *KIF20A* silencing significantly impaired the proliferative and invasive capabilities of ovarian cancer cells, and *FOXO1* could target *KIF20A* to manipulate cellular behaviors (Li et al., 2020). Xie et al. suggested that non-small cell lung cancer (NSCLC) had higher *KIF20A* expression than adjacent tissues. The deletion of the *KIF20A* remarkably alters cellular phenotypes, inhibits cell migration and invasion by reducing MMP-7 expression, as well as preventing cell proliferation by inactivating JNK signals, implying that *KIF20A* might function as a potential oncogene and a promising treatment target for NSCLC (Xie et al., 2020). In addition to pathological functions and therapeutic value, *KIF20A* has been recognized as an independent prognostic factor for various malignant cancers, such as bladder cancer, colorectal cancer, breast cancer, gastric cancer, epithelial ovarian cancer, and NSCLC (Ni et al., 2018; Sheng et al., 2018; Shen et al., 2019; Xiong et al., 2019; Nakamura et al., 2020). To our knowledge, no studies reported on the role of *KIF20A* as a prognostic indicator for LUAD patients.

Furthermore, a growing body of studies demonstrated that cancer cells, to some extent, shaped their surrounding microenvironment by secreting various cytokines and chemokines (Wu and Dai, 2017; Hinshaw and Shevde, 2019). The functional microenvironment would reprogram the surrounding cells, especially immune cells, and prompt them to take part in the tumor initiation and progression (Frankel et al., 2017). Additionally, the innate and adaptive immune cells in TME elicited crucial roles in tumor survival and maintenance (Gajewski et al., 2013; Cassim and Pouyssegur, 2019). In this study, we found that M0 macrophages and activated memory CD4^+^ T cells were not only significantly associated with *KIF20* expression but also negatively correlated with the survival of LUAD patients, indicating their potential actions in LUAD pathogenesis. Macrophages (Mφs) could polarize into inflammatory M1 Mφs or immune-suppressive M2 Mφs, of which TME preferred M2 Mφs compared with M1 Mφs (Genin et al., 2015; Nielsen and Schmid, 2017). Besides, Mφs were reported to foster the migration and invasion of cancer cells mediated by secreting epidermal growth factors (Elbaz et al., 2015; Zeng et al., 2019). High M0 Mφs infiltration was found in many solid tumors and commonly indicated unfavorable survival, such as colorectal cancer, digestive system cancer, and breast cancer (Bense et al., 2016; Ge et al., 2019; Yang et al., 2019). CD4^+^ central memory T cells mainly act on immune memory and immunoprotection during tumor metastasis (Bhattacharyya et al., 2010). In general, high factions of activated memory CD4^+^ T cells were correlated with good survival in esophageal cancer (Wang et al., 2020), which as opposite to our findings. However, the pathogenesis of TME involved in cancer progression is still not fully understood. Further research on pathogenesis is recommended in order to develop suitable therapeutic interventions.

The work contributed to the understanding of LUAD pathogenesis and the possible development of novel therapeutic strategies. However, the study has some limitations that need to be acknowledged. First of all, it was difficult to identify the specific molecular mechanisms of *KIF20A* in the development and progression of LUAD, highlighting the need for further studies to understand the underlying mechanism. Moreover, the inhibitory effects of KIF20A against cancer cell proliferation were not validated *in vivo*. Finally, the association of *KIF20A* with the TME, especially TICs communities, was not verified experimentally. In conclusion, *KIF20A* could be used as a reliable prognostic and therapeutic biomarker for LUAD. High *KIF20A* expression indicated worse OS, PFS, and DFS in LUAD patients. Knockdown of *KIF20A* efficiently suppressed cell proliferation, induced G2/M phase arrest, and promoted cellular apoptosis. However, more research is required to further validate the findings of this study.

## Data Availability

The original contributions presented in the study are included in the article/Supplementary Material, further inquiries can be directed to the corresponding author.

## References

[B1] Bense R. D. Sotiriou C. Piccart-Gebhart M. J. Haanen J. B. A. G. van Vugt M. A. T. M. de Vries E. G. E. (2016). Relevance of tumor-infiltrating immune cell composition and functionality for disease outcome in breast cancer. J. Natl. Cancer Inst. 109, djw192. 10.1093/jnci/djw192 27737921 PMC6284248

[B2] Bhattacharyya S. Md Sakib Hossain D. Mohanty S. Sankar Sen G. Chattopadhyay S. Banerjee S. (2010). Curcumin reverses T cell-mediated adaptive immune dysfunctions in tumor-bearing hosts. Cell. Mol. Immunol. 7, 306–315. 10.1038/cmi.2010.11 20305684 PMC4003225

[B3] Cassim S. Pouyssegur J. (2019). Tumor microenvironment: A metabolic player that shapes the immune response. Int. J. Mol. Sci. 21, 157. 10.3390/ijms21010157 31881671 PMC6982275

[B4] Chuang J. C. Stehr H. Liang Y. Das M. Diehn M. Huang J. (2017). ERBB2-Mutated metastatic non-small cell lung cancer: Response and resistance to targeted therapies. J. Thorac. Oncol. 12, 833–842. 10.1016/j.jtho.2017.01.023 28167203 PMC5402884

[B5] Elbaz M. Nasser M. W. Ravi J. Wani N. A. Ahirwar D. K. Zhao H. (2015). Modulation of the tumor microenvironment and inhibition of EGF/EGFR pathway: Novel anti-tumor mechanisms of cannabidiol in breast cancer. Mol. Oncol. 9, 906–919. 10.1016/j.molonc.2014.12.010 25660577 PMC4387115

[B6] Farhood B. Najafi M. Mortezaee K. (2019). CD8+ cytotoxic T lymphocytes in cancer immunotherapy: A review. J. Cell. Physiol. 234, 8509–8521. 10.1002/jcp.27782 30520029

[B7] Fontijn R. D. Goud B. Echard A. Jollivet F. van Marle J. Pannekoek H. (2001). The human kinesin-like protein RB6K is under tight cell cycle control and is essential for cytokinesis. Mol. Cell. Biol. 21, 2944–2955. 10.1128/mcb.21.8.2944-2955.2001 11283271 PMC86922

[B8] Frankel T. Lanfranca M. P. Zou W. (2017). The role of tumor microenvironment in cancer immunotherapy. Adv. Exp. Med. Biol. 1036, 51–64. 10.1007/978-3-319-67577-0_4 29275464

[B9] Fu L. Wang H. Wei D. Wang B. Zhang C. Zhu T. (2020). The value of CEP55 gene as a diagnostic biomarker and independent prognostic factor in LUAD and LUSC. PLoS One 15, e0233283. 10.1371/journal.pone.0233283 32437446 PMC7241791

[B10] Gajewski T. F. Schreiber H. Fu Y. X. (2013). Innate and adaptive immune cells in the tumor microenvironment. Nat. Immunol. 14, 1014–1022. 10.1038/ni.2703 24048123 PMC4118725

[B11] Gao Y. Yang J. Cai Y. Fu S. Zhang N. Fu X. (2018). IFN-γ-mediated inhibition of lung cancer correlates with PD-L1 expression and is regulated by PI3K-AKT signaling. Int. J. Cancer 143, 931–943. 10.1002/ijc.31357 29516506

[B12] Ge P. Wang W. Li L. Zhang G. Gao Z. Tang Z. (2019). Profiles of immune cell infiltration and immune-related genes in the tumor microenvironment of colorectal cancer. Biomed. Pharmacother. 118, 109228. 10.1016/j.biopha.2019.109228 31351430

[B13] Genin M. Clement F. Fattaccioli A. Raes M. Michiels C. (2015). M1 and M2 macrophages derived from THP-1 cells differentially modulate the response of cancer cells to etoposide. BMC Cancer 15, 577. 10.1186/s12885-015-1546-9 26253167 PMC4545815

[B14] Hinshaw D. C. Shevde L. A. (2019). The tumor microenvironment innately modulates cancer progression. Cancer Res. 79, 4557–4566. 10.1158/0008-5472.can-18-3962 31350295 PMC6744958

[B15] Hou J. Aerts J. den Hamer B. van Ijcken W. den Bakker M. Riegman P. (2010). Gene expression-based classification of non-small cell lung carcinomas and survival prediction. PLoS One 5, e10312. 10.1371/journal.pone.0010312 20421987 PMC2858668

[B16] Hou X. M. Zhang T. Da Z. Wu X. a. (2019). CHPF promotes lung adenocarcinoma proliferation and anti-apoptosis via the MAPK pathway. Pathology - Res. Pract. 215, 988–994. 10.1016/j.prp.2019.02.005 30826152

[B17] kayama H. Kohno T. Ishii Y. Shimada Y. Shiraishi K. Iwakawa R. (2012). Identification of genes upregulated in ALK-positive and EGFR/KRAS/ALK-negative lung adenocarcinomas. Cancer Res. 72, 100–111. 10.1158/0008-5472.can-11-1403 22080568

[B18] Landi M. T. Dracheva T. Rotunno M. Figueroa J. D. Liu H. Dasgupta A. (2008). Gene expression signature of cigarette smoking and its role in lung adenocarcinoma development and survival. PLoS One 3, e1651. 10.1371/journal.pone.0001651 18297132 PMC2249927

[B19] Li X. Shu K. Wang Z. Ding D. (2019). Prognostic significance of KIF2A and KIF20A expression in human cancer: A systematic review and meta-analysis. Med. Baltim. 98, e18040. 10.1097/md.0000000000018040 PMC6867763 31725680

[B20] Li Y. Guo H. Wang Z. Bu H. Wang S. Wang H. (2020). Cyclin F and KIF20A, FOXM1 target genes, increase proliferation and invasion of ovarian cancer cells. Exp. Cell Res. 395, 112212. 10.1016/j.yexcr.2020.112212 32771525

[B21] Ma Q. Wu K. Li H. Li H. Zhu Y. Hu G. (2019). ONECUT2 overexpression promotes RAS-driven lung adenocarcinoma progression. Sci. Rep. 9, 20021. 10.1038/s41598-019-56277-2 31882655 PMC6934839

[B22] Nakamura M. Takano A. Thang P. M. Tsevegjav B. Zhu M. Yokose T. (2020). Characterization of KIF20A as a prognostic biomarker and therapeutic target for different subtypes of breast cancer. Int. J. Oncol. 57, 277–288. 10.3892/ijo.2020.5060 32467984

[B23] Ni M. Liu X. Wu J. Zhang D. Tian J. Wang T. (2018). Identification of candidate biomarkers correlated with the pathogenesis and prognosis of non-small cell lung cancer via integrated bioinformatics analysis. Front. Genet. 9, 469. 10.3389/fgene.2018.00469 30369945 PMC6194157

[B24] Nielsen S. R. Schmid M. C. (2017). Macrophages as key drivers of cancer progression and metastasis. Mediat. Inflamm. 2017, 1–11. 10.1155/2017/9624760 PMC5292164 28210073

[B25] Schabath M. B. Welsh E. A. Fulp W. J. Chen L. Teer J. K. Thompson Z. J. (2016). Differential association of STK11 and TP53 with KRAS mutation-associated gene expression, proliferation and immune surveillance in lung adenocarcinoma. Oncogene 35, 3209–3216. 10.1038/onc.2015.375 26477306 PMC4837098

[B26] Selamat S. A. Chung B. S. Girard L. Zhang W. Zhang Y. Campan M. (2012). Genome-scale analysis of DNA methylation in lung adenocarcinoma and integration with mRNA expression. Genome Res. 22, 1197–1211. 10.1101/gr.132662.111 22613842 PMC3396362

[B27] Shan L. Zhao M. Lu Y. Ning H. Yang S. Song Y. (2019). CENPE promotes lung adenocarcinoma proliferation and is directly regulated by FOXM1. Int. J. Oncol. 55, 257–266. 10.3892/ijo.2019.4805 31115500

[B28] Shen T. Yang L. Zhang Z. Jianpeng Y. Dai L. Gao M. (2019). KIF20A affects the prognosis of bladder cancer by promoting the proliferation and metastasis of bladder cancer cells. Dis. Markers 2019, 1–9. 10.1155/2019/4863182 PMC6481133 31093305

[B29] Sheng Y. Wang W. Hong B. Jiang X. Sun R. Yan Q. (2018). Upregulation of KIF20A correlates with poor prognosis in gastric cancer. Cancer Manag. Res. 10, 6205–6216. 10.2147/cmar.s176147 30538567 PMC6260125

[B30] Su L. J. Chang C. W. Wu Y. C. Chen K. C. Lin C. J. Liang S. C. (2007). Selection of DDX5 as a novel internal control for Q-RT-PCR from microarray data using a block bootstrap re-sampling scheme. BMC Genomics 8, 140. 10.1186/1471-2164-8-140 17540040 PMC1894975

[B31] Sun G. Z. Zhao T. W. (2019). Lung adenocarcinoma pathology stages related gene identification. Math. Biosci. Eng. 17, 737–746. 10.3934/mbe.2020038 31731374

[B32] Wang L. Wei Q. Zhang M. Chen L. Li Z. Zhou C. (2020). Identification of the prognostic value of immune gene signature and infiltrating immune cells for esophageal cancer patients. Int. Immunopharmacol. 87, 106795. 10.1016/j.intimp.2020.106795 32707495

[B33] Wculek S. K. Cueto F. J. Mujal A. M. Melero I. Krummel M. F. Sancho D. (2020). Dendritic cells in cancer immunology and immunotherapy. Nat. Rev. Immunol. 20, 7–24. 10.1038/s41577-019-0210-z 31467405

[B34] Wu T. Dai Y. (2017). Tumor microenvironment and therapeutic response. Cancer Lett. 387, 61–68. 10.1016/j.canlet.2016.01.043 26845449

[B35] Xie F. He C. Gao S. Yang Z. Li L. Qiao L. (2020). *Retracted:*KIF20A silence inhibits the migration, invasion and proliferation of non‐small cell lung cancer and regulates the JNK pathway. Clin. Exp. Pharmacol. Physiol. 47, 135–142. 10.1111/1440-1681.13183 31557334

[B36] Xiong M. Zhuang K. Luo Y. Lai Q. Luo X. Fang Y. (2019). KIF20A promotes cellular malignant behavior and enhances resistance to chemotherapy in colorectal cancer through regulation of the JAK/STAT3 signaling pathway. Aging (Albany NY) 11, 11905–11921. 10.18632/aging.102505 31841120 PMC6949076

[B37] Yang S. Liu T. Cheng Y. Bai Y. Liang G. (2019). Immune cell infiltration as a biomarker for the diagnosis and prognosis of digestive system cancer. Cancer Sci. 110, 3639–3649. 10.1111/cas.14216 31605436 PMC6890448

[B38] Yao S. Fan L. Y. Lam E. W. (2018). The FOXO3-FOXM1 axis: A key cancer drug target and a modulator of cancer drug resistance. Semin. Cancer Biol. 50, 77–89. 10.1016/j.semcancer.2017.11.018 29180117 PMC6565931

[B39] Zeng X. Y. Xie H. Yuan J. Jiang X. Y. Yong J. H. Zeng D. (2019). M2-like tumor-associated macrophages-secreted EGF promotes epithelial ovarian cancer metastasis via activating EGFR-ERK signaling and suppressing lncRNA LIMT expression. Cancer Biol. Ther. 20, 956–966. 10.1080/15384047.2018.1564567 31062668 PMC6606001

[B40] Zhan P. Zhang B. Xi G. M. Wu Y. Liu H. b. Liu Y. f. (2017). PRC1 contributes to tumorigenesis of lung adenocarcinoma in association with the Wnt/β-catenin signaling pathway. Mol. Cancer 16, 108. 10.1186/s12943-017-0682-z 28646916 PMC5483280

[B41] Zhang Q. Di J. Ji Z. Mi A. Li Q. Du X. (2020). KIF20A predicts poor survival of patients and promotes colorectal cancer tumor progression through the JAK/STAT3 signaling pathway. Dis. Markers 2020, 1–11. 10.1155/2020/2032679 PMC7368235 32695240

[B42] Zhang Z. Chai C. Shen T. Li X. Li C. Ji J. (2019). Aberrant KIF20A expression is associated with adverse clinical outcome and promotes tumor progression in prostate cancer. Dis. Markers 2019, 1–10. 10.1155/2019/4782730 PMC6745134 31565099

[B43] Zhao X. Li X. Zhou L. Ni J. Yan W. Ma R. (2018). LncRNA HOXA11-AS drives cisplatin resistance of human LUAD cells via modulating miR-454-3p/Stat3. Cancer Sci. 109, 3068–3079. 10.1111/cas.13764 30099826 PMC6172072

[B44] Zhu K. Liu Q. Zhou Y. Tao C. Zhao Z. Sun J. (2015). Oncogenes and tumor suppressor genes: Comparative genomics and network perspectives. BMC Genomics 16, S8. 10.1186/1471-2164-16-s7-s8 PMC4474543 26099335

