# Supplementary material for: An evaluation of KIF20A as a prognostic factor and therapeutic target for lung adenocarcinoma using integrated bioinformatics analysis
Source: Front Bioeng Biotechnol. 2022 Dec 23;10:993820. doi: 10.3389/fbioe.2022.993820 (PMC9816395; doi:10.3389/fbioe.2022.993820)
Supplement: Supplementary file 1 [file DataSheet1.ZIP › SupplementaryFiles.docx]

**
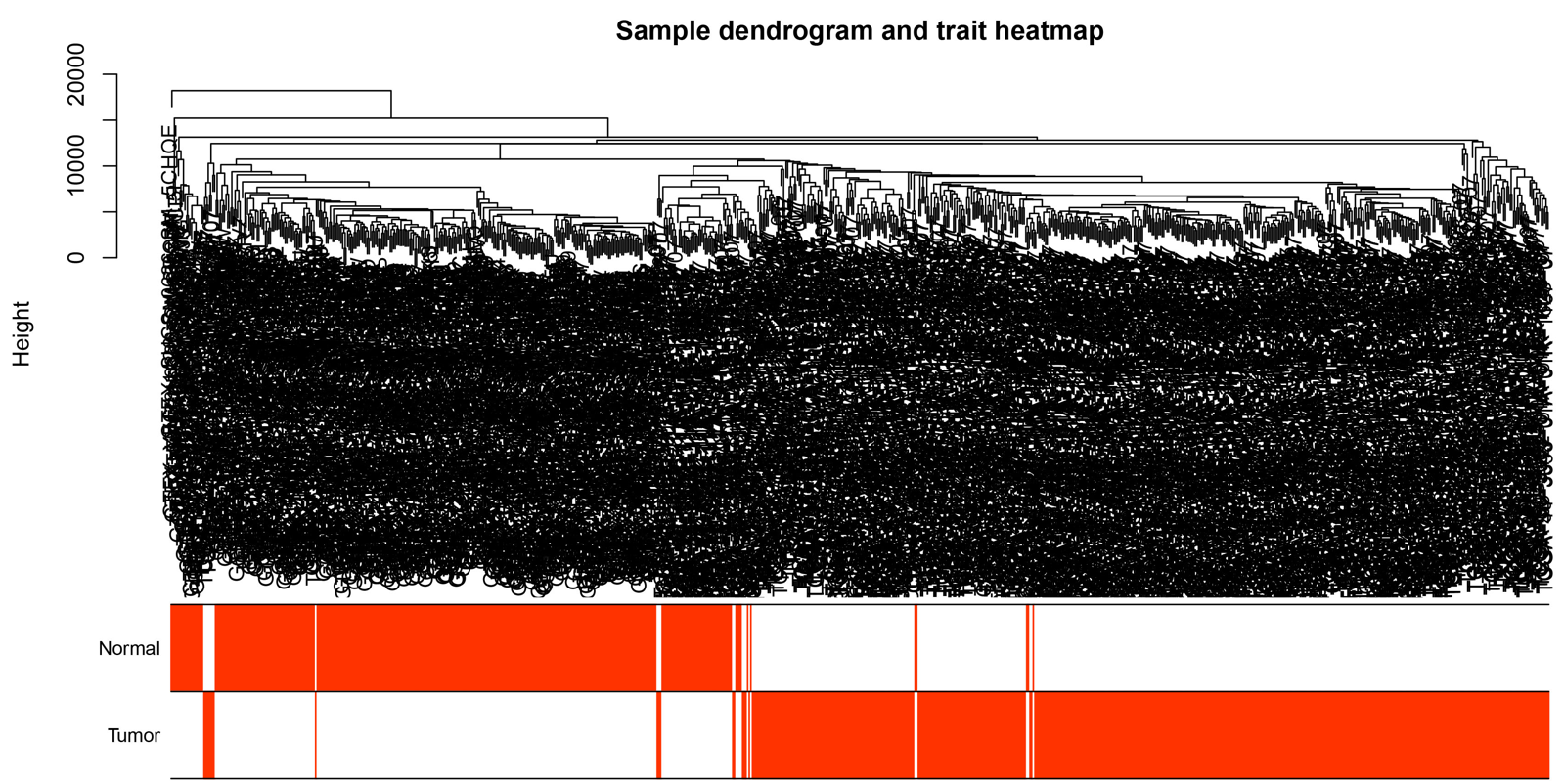
**

**Supplementary Figure 1.** Sample clustering dendrogram and clinical traits indicator based on the expression data from the GTEx and TCGA datasets of normal lung samples (n=342) and LUAD samples (n=497). The red color block corresponds to the clinical information of samples in the clustering diagram.


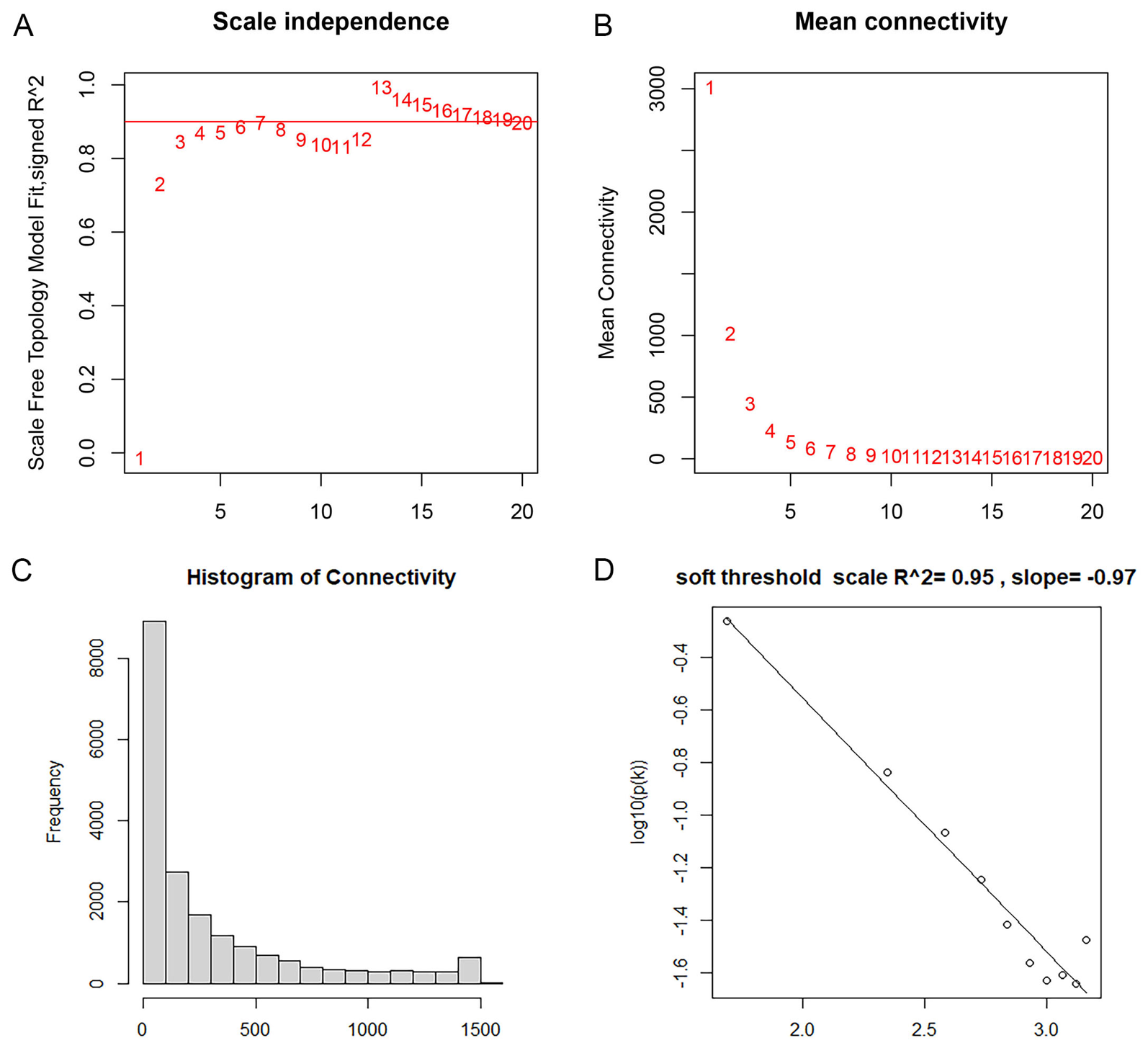


**Supplementary Figure 2.** **Determination of soft-thresholding power in the weighted gene co-expression network analysis (WGCNA).** Topology analysis of the scale-free fit index (A) and the mean connectivity (B) for different soft-thresholding powers (*β*). Histogram of connectivity distribution (C) and scatter plot for checking the scale free topology (D) when *β* = 5.


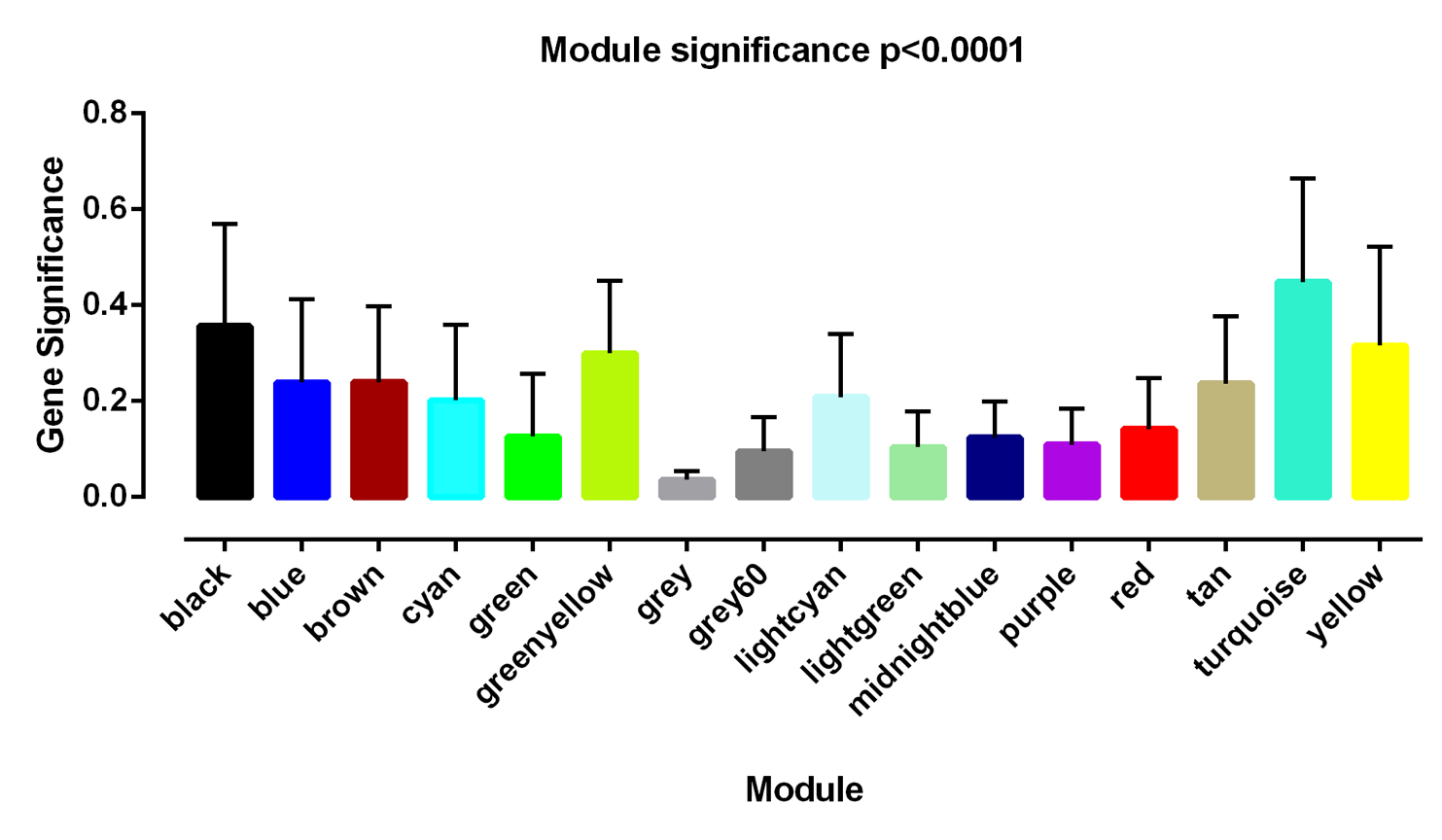


**Supplementary Figure 3.** Histogram for module significance illustrating the distribution of the average gene significance and module errors within the tumor.


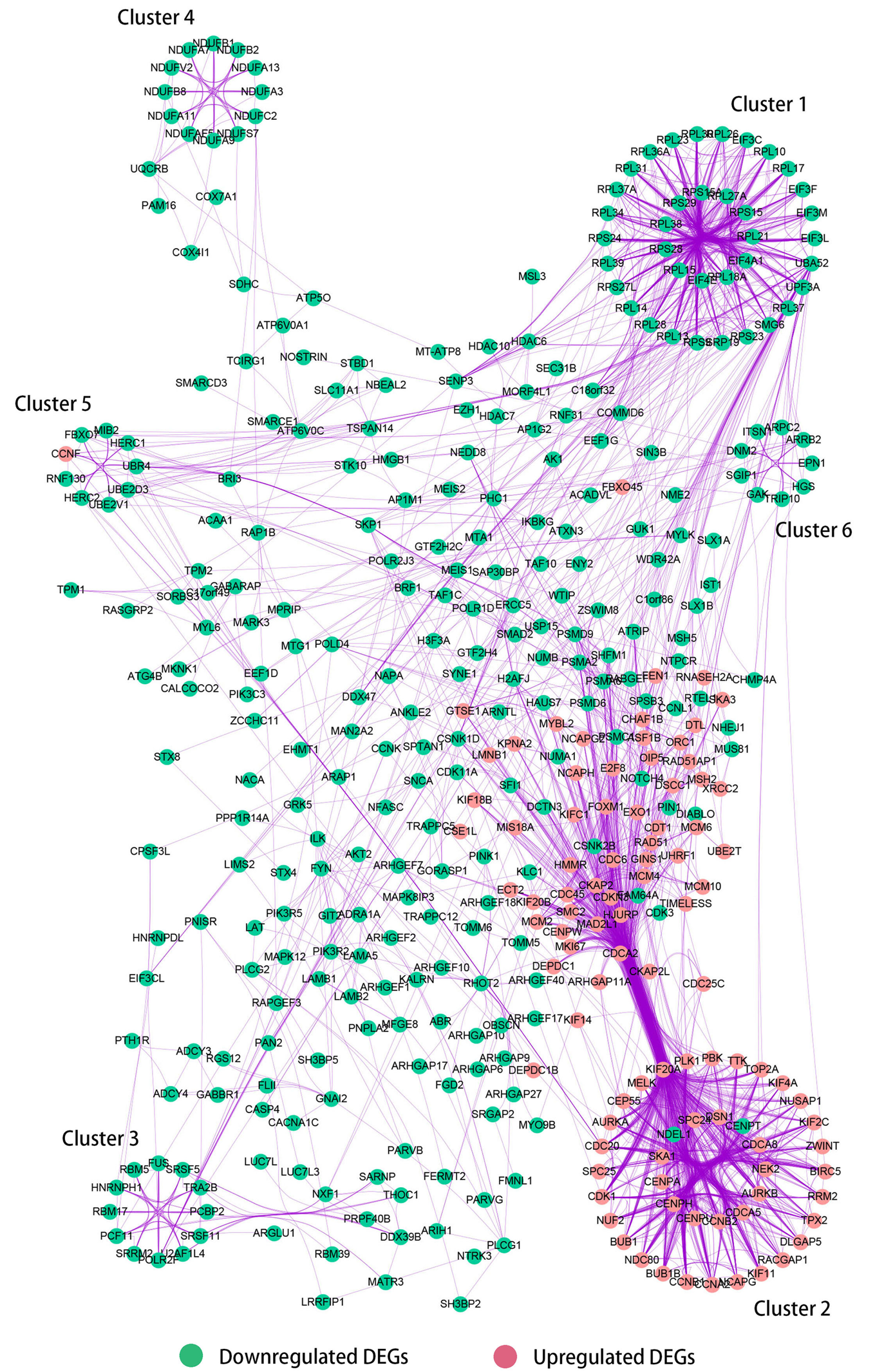


**Supplementary Figure 4. Construction of protein-protein interaction (PPI) network.** PPI network and clustering analysis for the 904 common differentially expressed genes (DEGs). The green and red colored spots represent the downregulated and upregulated DEGs, respectively.


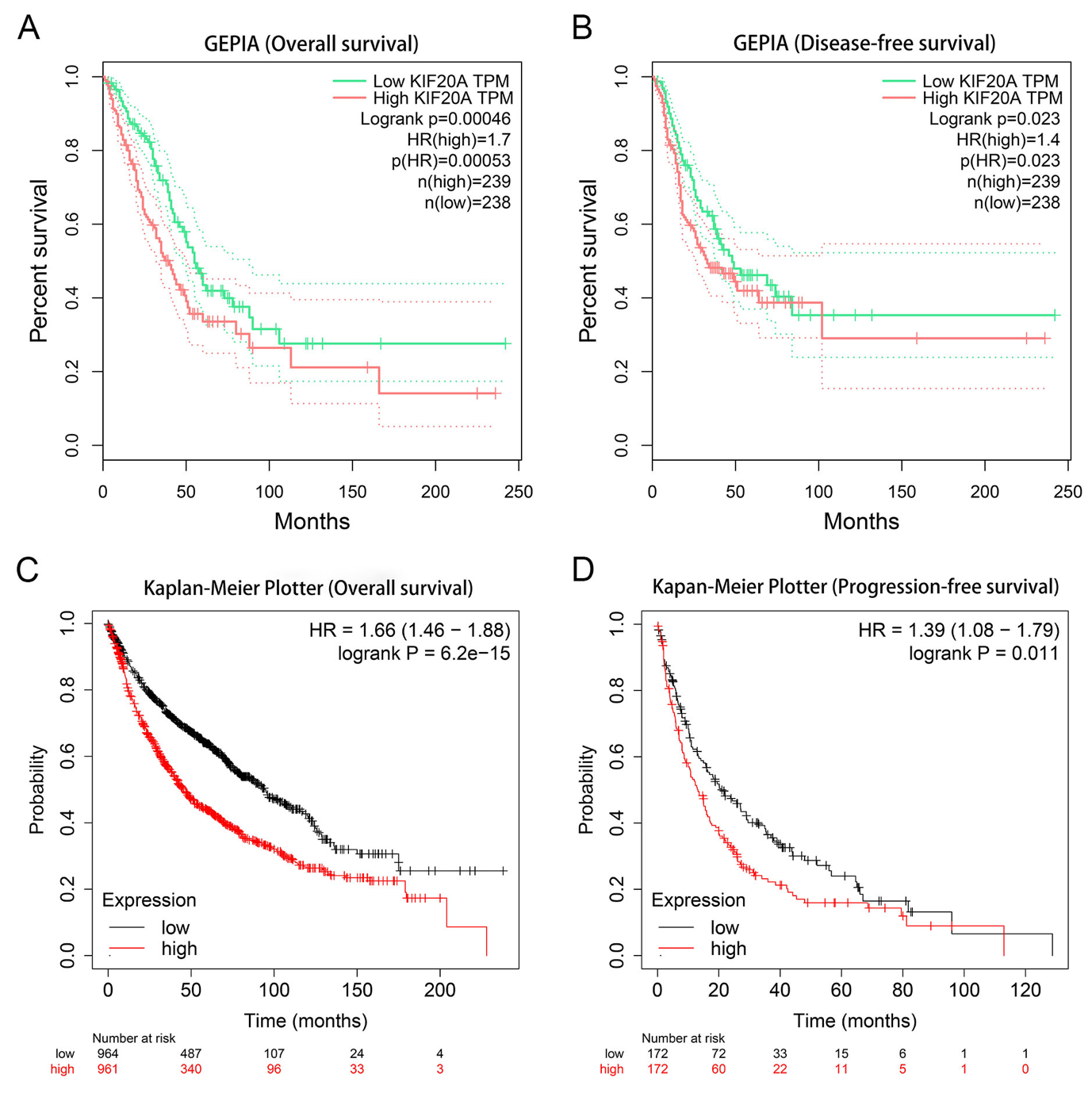


**Supplementary Figure 5. Survival analysis of *KIF20A* in GEPIA and Kaplan-Meier Plotter platforms.** High *KIF20A* expression was negatively associated with overall survival (OS) (A) and disease-free survival (DFS) (B) in the GEPIA database. High *KIF20A* expression significantly indicated poor OS (C) and progression-free survival (PFS) (D) in the Kaplan-Meier Plotter platform.


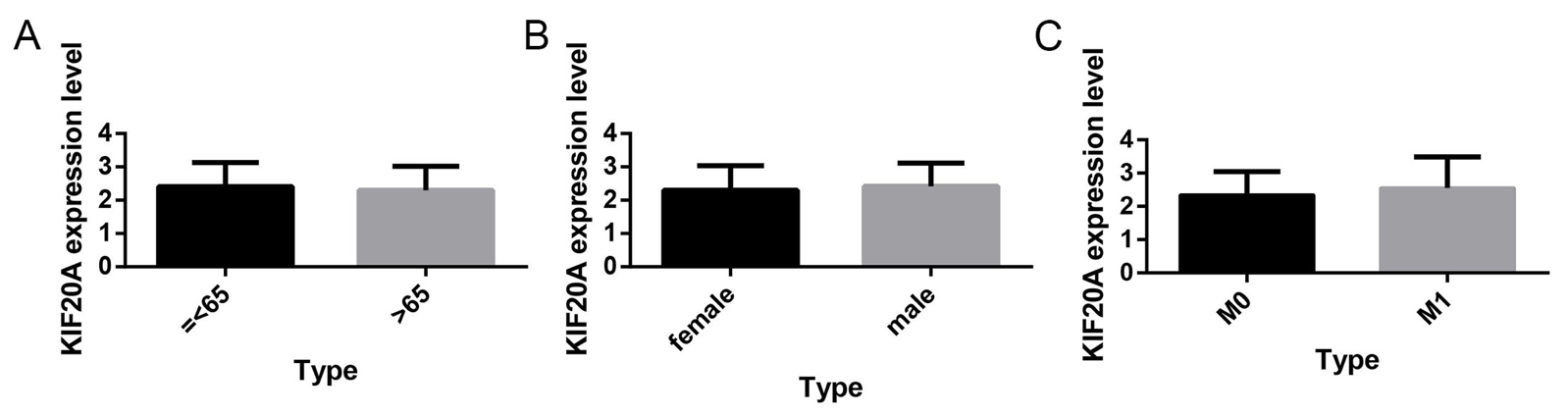


**Supplementary Figure 6. Correlation of *KIF20A* expression with age, gender, and M stage in lung** [**adenocarcinoma**](javascript:;) **(LUAD).** The histogram shows that KIF20A expression is not influenced by age (A), gender (B) or M classification (C).


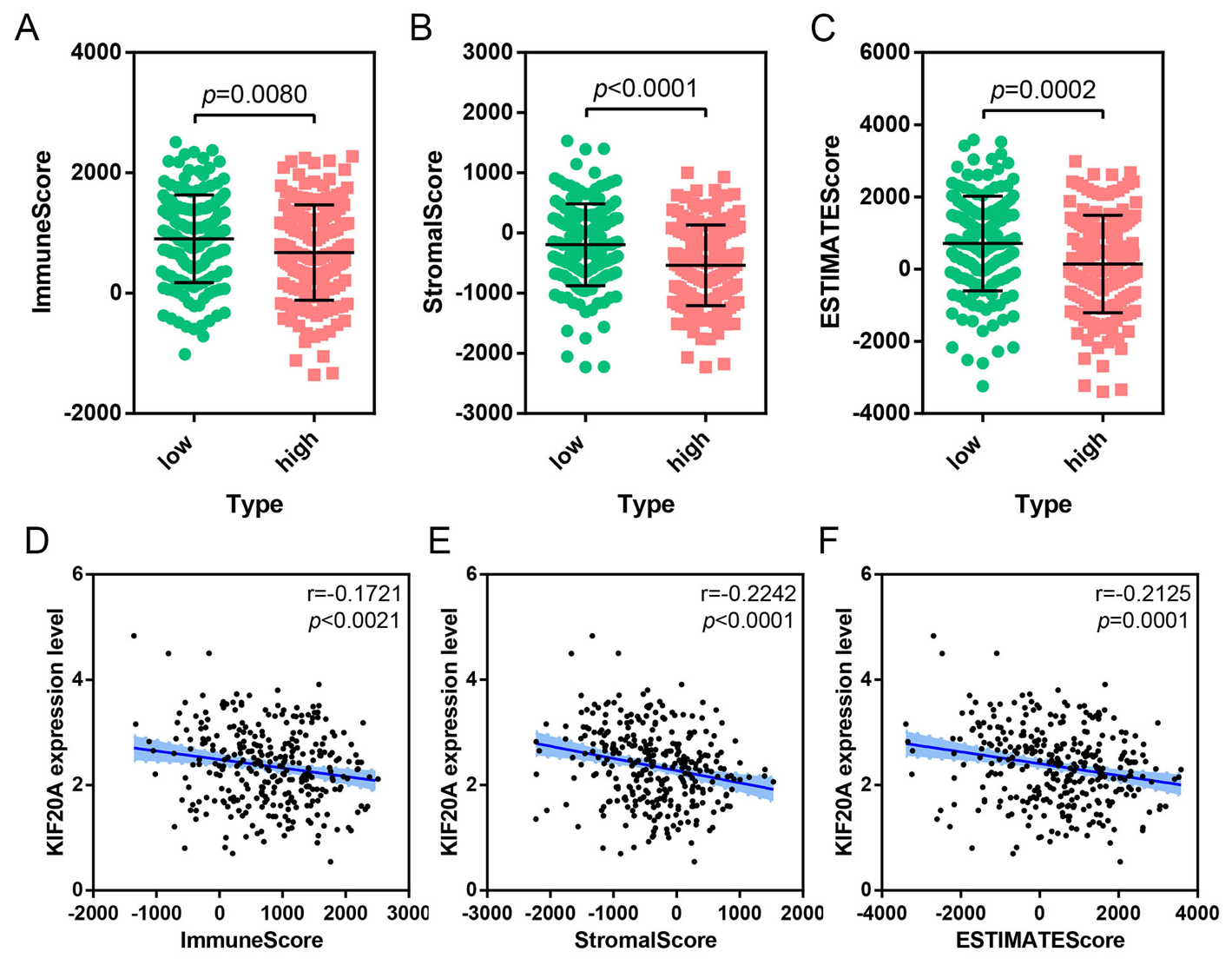


**Supplementary Figure 7. Correlation of *KIF20A* expression with tumor microenvironment scores.** Difference analysis of the immune score (A), stromal score (B), and ESTIMATE score (C) between low and high *KIF20A* expression subgroups determined by the median *KIF20A* expression level. Correlation analysis of *KIF20A* expression with the immune score (D), stromal score (E), and ESTIMATE score (F).


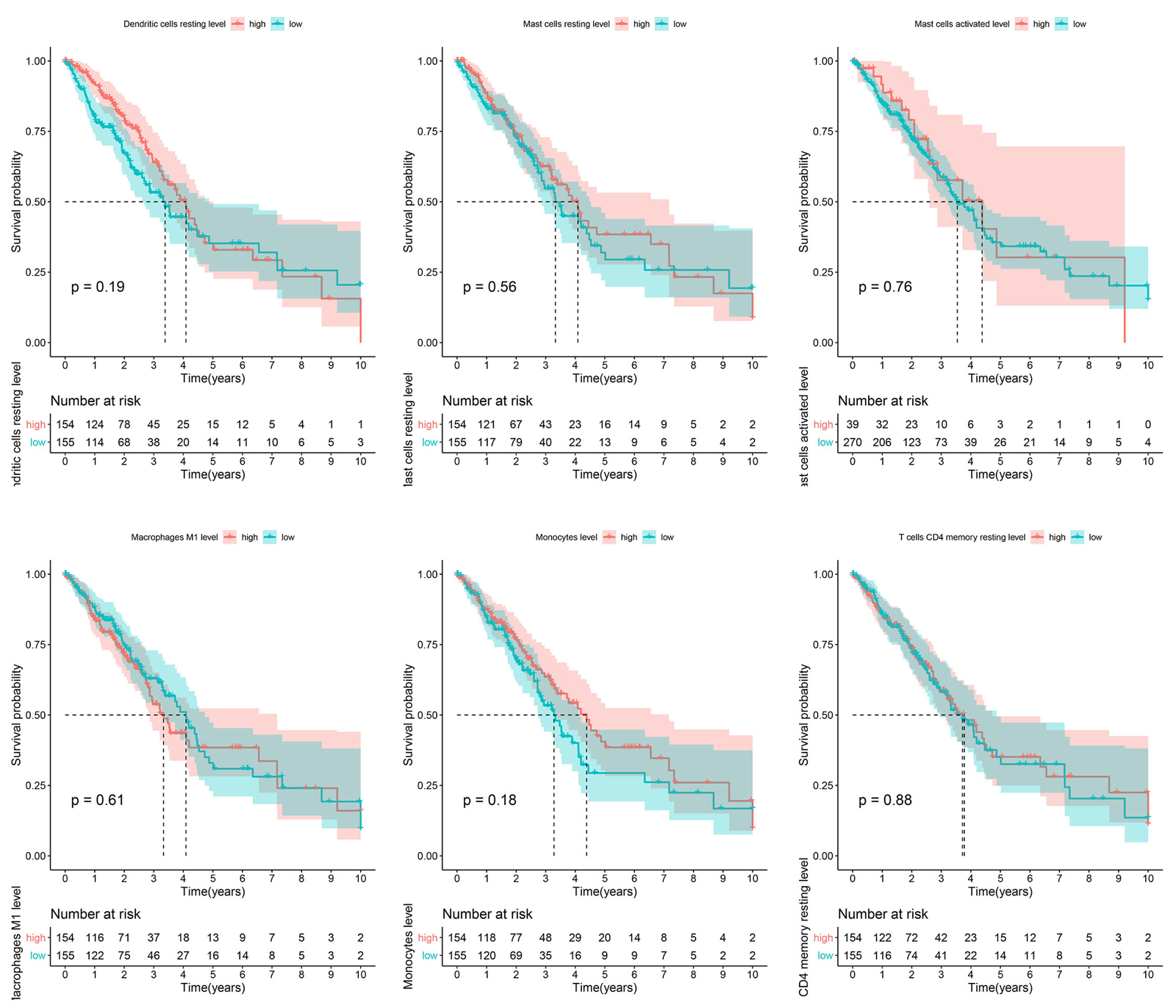


**Supplementary Figure 8.** **Survival analysis for the six different types of tumor-infiltrating immune cells (TICs) in LUAD.**
